# Comprehensive genome-wide identification of the NPF gene family and functional characterization of *GmNPF6.8* regulating root development in soybean

**DOI:** 10.1186/s12870-026-08559-x

**Published:** 2026-03-17

**Authors:** Wei Wang, Jiajia Li, Xiaonan Zhao, Xiaoyu Hu, Guoji Wang, Qinqin Zhang, Haowei Zheng, Pengyu Bai, Long Miao, Xiaobo Wang

**Affiliations:** School of Agronomy, Anhui Agricultural University, Hefei, 230036 China

**Keywords:** Expression profile, *GmNPF6.8*, Low nitrogen stress, Nitrate transporter 1/peptide transporter family, Soybean

## Abstract

**Background:**

The nitrate transporter 1/peptide transporter family (NPF) plays a key role in nitrate uptake, transport, and nitrogen use efficiency in plants. Although *NPF* genes have been widely studied in many species, their genomic organization, evolutionary patterns, and functional roles in soybean remain unclear. Soybean is an important legume with high nitrogen demand and the ability to fix atmospheric nitrogen through symbiosis.

**Results:**

In this study, 126 *GmNPF* genes were identified in the Wm82.a4.v1 genome. These genes were classified into 8 subfamilies and were unevenly distributed across 19 chromosomes. Family expansion was mainly driven by segmental duplication. Ka/Ks analysis indicated strong purifying selection. Promoter analysis revealed cis-regulatory elements associated with light response, phytohormone signaling, and abiotic stress. Expression profiling across tissues showed clear spatial and temporal patterns for 112 *GmNPF* genes. *GmNPF6.8* was predominantly expressed in roots. Under low-nitrogen conditions, many *GmNPF* genes were differentially expressed. *GmNPF5.13*, *GmNPF5.5*, *GmNPF7.13*, *GmNPF7.12*, *GmNPF7.14*, and *GmNPF2.11* were significantly upregulated, whereas *GmNPF6.8* and *GmNPF6.9* were significantly downregulated in soybean roots. Genetic diversity analysis of *GmNPF6.8* in 4,068 soybean accessions identified 3 coding-region haplotypes. *GmNPF6.8*^*Hap1*^ showed clear evidence of strong artificial selection. Subcellular localization assays confirmed that *GmNPF6.8* is localized to the plasma membrane. Overexpression of *GmNPF6.8* in *Arabidopsis* and soybean hairy roots significantly reduced root length and root density. It also altered the expression of key genes involved in root development. Further analysis showed that *GmARF11* directly binds to the promoter of *GmNPF6.8* and represses its transcription.

**Conclusions:**

This study clarified the genomic and evolutionary features of the *GmNPF* family and identified *GmNPF6.8* as a negative regulator of root development. These findings provide a potential target for improving nitrogen use efficiency in soybean breeding.

**Supplementary Information:**

The online version contains supplementary material available at 10.1186/s12870-026-08559-x.

## Background

Nitrogen (N) is an essential mineral nutrient for plants and is required in large amounts. It is a key component of nucleotides, proteins, chlorophyll, and plant hormones. Crops mainly absorb nitrogen from the soil in the form of nitrate and ammonium, both of which are critical for plant growth and development [1, 2]. Nitrate uptake and transport are mediated by four major protein families: the nitrate transporter 1/peptide transporter family (NPF), the nitrate transporter 2 (NRT2), the chloride ion channel family (CLC), and the slowly activating anion channel family (SLAC/SLAH) [3, 4]. The NPF family is one of the largest transporter families in plants. Its members play diverse roles in nitrate uptake and distribution throughout the plant. Nitrate is the primary form of nitrogen absorbed and utilized by plants, and its uptake and long-distance transport largely depend on NPF transporters [5].

The NPF gene family encodes transporter proteins involved in the uptake and transport of nitrogen compounds and peptides. These proteins play important roles in plant growth, development, and stress responses. Previous studies have classified NPF genes into 8 subfamilies [6]. NPF members have been identified in various plants, such as *Arabidopsis thaliana* [7], *Oryza sativa* [8], *Triticum aestivum* [9], *Zea mays* L. [10, 11], *Brassica napus* L. [12], and *Gossypium* spp [13]. Their identification has facilitated functional studies of plant NPF transporters. Functional analyses show that NPF proteins are mainly involved in plant growth and development, nitrogen uptake, and stress responses. *AtNPF6.3/AtNRT1.1* was the first nitrate transporter identified in *Arabidopsis thaliana*. It regulates the transport of nitrate from the root system to NO_3_^−^ uptake in plants, and playing a dual affinity role in nitrate transport within both high and low affinity ranges [14, 15]. *AtNPF8.2* promotes peptide transport to germinating pollen and may also function in mature pollen, ovules, and seeds. Overexpression promotes aboveground growth and increases nitrogen content level [16]. In rice, *OsNRT1.1 A/OsNPF6.3* belongs to the NPF family. Its overexpression significantly improves nitrogen use efficiency and yield, and promotes early maturation [17]. Overexpression of *OsNPF7.1* or knockout of *OsNPF7.4* significantly increases biomass, tiller number, grain filling, and yield [18]. Overexpression of *OsNPF7.6* increases tiller number per plant, grain weight per panicle, and increased grain yield and nitrogen utilization efficiency [19]. *zmnpf7.9* leads to delayed endosperm development, abnormal starch accumulation, and reduced 100-grain weight in maize, indicating that *ZmNPF7.9* plays a specific role in seed development and grain weight by regulating nutrient transport and metabolism [20]. Silencing *GhNPF6.14* through virus-induced gene silencing affects cotton growth and reduces nitrogen uptake and accumulation [13]. Previous studies showed that *AtNPF2.3* is mainly expressed in the root stele under stress conditions. It mediates nitrate loading into the root xylem sap and may contribute to adaptation to mild saline-alkali stress [21]. *AtNPF2.9/NRT1.9* is a low-affinity nitrate transporter that mediates nitrate loading into the root phloem. It influences nitrate distribution between roots and shoots [22]. *AtNPF2.5* is primarily expressed in root cortical cells, and its transcription is regulated by salt stress [23]. In rice, *OsNPF7.9* encodes a low-affinity nitrate transporter that is preferentially expressed in the xylem parenchyma cells. It plays a key role in balancing growth and stress tolerance [24]. *AtNPF6.3* has also been linked to tolerance to various environmental stresses, including Cd^2+^, H^+^, Pb^2+^, Zn^2+^, drought, and salinity [25]. Together, these findings highlight the essential roles of NPF transporters in plant growth, development, and stress responses.

Soybean (*Glycine max* [L.] Merr.) is a highly versatile crop used as a raw material for food production, animal feed production, the manufacture of industrial products and other applications [26]. Its yield is often limited by nitrogen availability. Although the NPF family has been studied in several species, the functions and evolutionary history of the *GmNPF* genes remain unclear. In this study, we performed a comprehensive bioinformatic analysis of the *GmNPF* family, including gene structure, conserved motifs, phylogenetic relationships, duplication events, chromosomal distribution, and cis-regulatory elements. We also conducted a comparative phylogenetic analysis using *NPF* genes from dicot species (*Arabidopsis thaliana*, *Vigna unguiculata*, and *Solanum lycopersicum*) and the monocot species *Oryza sativa.* This analysis helped clarify the evolutionary history of the NPF family in soybean. The expression patterns of *GmNPF* genes in different tissues were analyzed to clarify its spatiotemporal expression characteristics and identify potential tissue-specific functional members. RNA-seq analysis was then performed on soybean roots under low-nitrogen conditions to identify core *GmNPF* genes responsive to nitrogen deficiency. This approach also provided insight into their potential roles in adaptation to low nitrogen. Among these genes, we focused on *GmNPF6.8*, which is specifically expressed in roots and is significantly downregulated under low-nitrogen conditions. Subcellular localization assays confirmed its cellular distribution. Ectopic overexpression in *Arabidopsis thaliana* and overexpression in transgenic soybean hairy roots revealed that *GmNPF6.8* negatively regulates root development under different nitrogen levels. In addition, population genetics analysis and experiments on *GmARF11*-mediated transcriptional regulation were conducted to clarify the molecular mechanism by which *GmNPF6.8* acts as a negative regulator of root development. Overall, this study provides new genetic resources and a theoretical basis for improving nitrogen use efficiency in soybean.

## Materials and methods

### Plant material and growth conditions

The soybean cultivars ‘Williams 82’ (W82) and ‘Tianlong No.1’ (TL1) were grown in a greenhouse at Anhui Agricultural University under controlled conditions: 28 °C, 70% relative humidity, and a 16 h light/8 h dark photoperiod. Seeds of the W82 variety were germinated and transferred to a modified Hoagland nutrient solution containing 4 mM CaCl_2_·2H_2_O, 2.5 mM K_2_SO_4_, 1 mM KH_2_PO_4_, 2.5 mM NH_4_NO_3_, 2 mM MgSO_4_, 0.08 mM FeNaEDTA, 5 µM KI, 0.1 mM H_3_BO_3_, 0.1 µM MnSO_4_·H_2_O, 30 µM ZnSO_4_·7H_2_O, 1 µM Na_2_MoO_4_·2H_2_O, 0.1 µM CuSO_4_·5H_2_O, and 0.1 µM CoCl_2_·6H_2_O (pH 5.8). Seedlings were grown hydroponically for 7 days, until the first trifoliate leaf was fully expanded. To induce low nitrogen (LN) stress, plants were transferred to a nutrient solution containing 10% of the original nitrogen concentration (0.25 mM NH_4_NO₃). They were grown for an additional 5 days in a growth chamber at 28 °C under a 16 h light/8 h dark photoperiod. Root samples were then collected, immediately frozen in liquid nitrogen, and stored at − 80 °C for RNA extraction and gene expression analysis. Wild-type (WT) *Arabidopsis thaliana* (Col-0), *GmNPF6.8*-overexpressing lines, and the *atnpf6.3/atnrt1.1* mutant lines were grown in a growth chamber at 25 °C under a 16 h light/8 h dark photoperiod.

### Identification of *NPF* genes in soybean

The soybean genome sequence (FASTA) and corresponding GFF3 annotation file (Wm82.a4.v1) were downloaded from the Phytozome database (https://phytozome-next.jgi.doe.gov/). To identify NPF family members in soybean, two complementary methods were used. First, a BLASTP search was conducted using 53 *Arabidopsis thaliana* NPF protein sequences as queries against the soybean protein database. Second, the Hidden Markov Model (HMM) profile of the Proton-dependent Oligopeptide Transporter 2 (PTR2) domain (PF00854) was retrieved from the Pfam database (http://pfam.xfam.org/). This profile was used to search the soybean protein dataset with HMMER 3.0 (hmmsearch) using the default threshold (E < 1 × 10^− 5^). The candidate sequences obtained from both approaches were combined, and redundant entries were removed. The presence of the conserved PTR2 domain in each candidate was further confirmed using SMART (http://smart.embl-heidelberg.de/), NCBI CD-Search, and the Pfam database. Sequences lacking the PTR2 domain were excluded from further analysis.

### Phylogenetic analysis

The evolutionary relationships among *GmNPF* family members were analyzed using *Arabidopsis thaliana* NPF protein sequences as references. Amino acid sequences were aligned using ClustalW with default settings. Following alignment, a phylogenetic tree was constructed in MEGA 7.0 using the maximum-likelihood method with 1,000 bootstrap replicates. The resulting phylogenetic tree was visualized and annotated using iTOL (https://itol.embl.de/).

### Chromosome localization and synteny analysis

The chromosomal positions of *GmNPF* genes were extracted from the soybean Wm82.a4.v1 genome annotation file (GFF3) and visualized using TBtools [27]. Gene density along each chromosome was calculated using a 100-kb sliding window and displayed as a color gradient from blue to red. For intra-genomic analysis, syntenic relationships among *GmNPF* genes were identified with MCScanX and visualized in TBtools.

For interspecific collinearity analysis, genome sequences and annotation files for *Arabidopsis thaliana* (TAIR10), *Vigna unguiculata* (V1.1), *Oryza sativa* (MSU7.0), and *Solanum lycopersicum* (ITAG3.2) were downloaded from Phytozome (https://phytozome-next.jgi.doe.g.ov/). Syntenic relationships between *GmNPF* genes and their orthologs in these species were identified and plotted using the Dual Synteny Plotter function in TBtools [27].

### Cis-regulatory element and conserved motif analyses of *GmNPF* genes

Cis-regulatory elements in the 2,000 bp upstream sequences of *GmNPF* genes were predicted using PlantCARE (https://bioinformatics.psb.ugent.be/webtools/plantcare/html/). Conserved amino acid motifs in GmNPF proteins were identified using MEME v5.5.4 (http://meme-suite.org/tools/meme), with a maximum of 10 motifs. The exon-intron structures of *GmNPF* genes were extracted from the GFF3 annotation file and visualized using TBtools [27].

### Expression profile analyses of *GmNPF* genes

Transcriptome data for *GmNPF* genes across different soybean tissues were retrieved from SoyBase (https://soybase.org/soyseq/) [28] and quantified as fragments per kilobase of exon per million mapped reads (FPKM). Data on *GmNPF* expression under low nitrogen stress were obtained from the Plant Public RNA-seq Database (PPRD, http://ipf.sustech.edu.cn/pub/plantrna/) [29]. Expression heatmaps were generated using Log₂(FPKM) values in TBtools. To investigate co-expression patterns, pairwise correlation coefficients were calculated from FPKM values using OmicShare online tools (https://www.omicshare.com/tools/). Phylogenetically clustered heatmaps were also constructed in TBtools [27].

### RNA extraction and qRT-PCR analysis

Total RNA was extracted from plant tissues using the TaKaRa MiniBEST Plant RNA Extraction Kit (TaKaRa, Dalian, China) and treated with RNase-free DNase I to remove genomic DNA contamination. First-strand cDNA was synthesized from 1 µg of total RNA using the PrimeScript RT reagent kit (TaKaRa). Gene-specific primers were designed with Primer Premier 5.0 and are listed in Supplementary Table S9. qRT-PCR was performed on a CFX96 Real-Time PCR System (Bio-Rad, USA) using SYBR Premix Ex Taq™ II (TaKaRa, Dalian, China). Each 25 µL reaction contained 12.5 µL SYBR Premix, 1 µL of each primer (10 µM), 2 µL of diluted cDNA, and 8.5 µL ddH₂O. The thermal cycling program was: 95 °C for 30 s, followed by 40 cycles of 95 °C for 5 s and 60 °C for 30 s. A melting curve analysis was performed to confirm amplification specificity. Relative gene expression levels were calculated using the 2^−ΔΔCt^ method [30]. *Atactin* (AT5G09810) and *Gmactin* (GenBank accession TC204137) were used as internal controls for *Arabidopsis* and soybean, respectively [31]. The experiment included three independent biological replicates, each with three technical replicates. Statistical analyses were performed using GraphPad Prism 5 [32].

### Subcellular localization of *GmNPF6.8*

The coding sequence of the *GmNPF6.8* was amplified from roots of the soybean cultivar W82 by RT-PCR using high-fidelity KOD-Plus DNA polymerase (TOYOBO, Osaka, Japan). The amplified sequence was cloned into the pHZM33 vector, which carries the CaMV35S promoter and a Green Fluorescent Protein (GFP) tag. Primers used for cloning are listed in Supplementary Table S9. The verified plasmid was co-transformed with the plasma membrane marker protein *AtLTI6B*-mCherry [33] into *Agrobacterium tumefaciens* strain GV3101. Bacterial cultures were resuspended in subcellular localization buffer containing 10 mM MES (pH 5.6), 10 mM MgCl₂, and 100 µM acetosyringone. The empty pHZM33-GFP vector in GV3101 served as a negative control. Agrobacterium suspensions were infiltrated into leaves of 5-week-old *Nicotiana benthamiana* plants using a syringe. The infiltrated leaves were incubated in a dark, sealed environment for 48 h. GFP and mCherry fluorescence signals were observed using a confocal laser scanning microscope (Leica, Germany) to determine the subcellular localization of *GmNPF6.8*.

### Transformation and overexpression of *GmNPF6.8* in *Arabidopsis thaliana*

The coding sequence of *GmNPF6.8* was amplified from roots of the soybean cultivar W82 by RT-PCR using KOD-Plus DNA polymerase (TOYOBO, Osaka, Japan) and cloned into the pCAMBIA1305 vector to generate the pCAMBIA1305:*GmNPF6.8* construct. All primers used for cloning are listed in Supplementary Table S9. The construct was introduced into *Agrobacterium tumefaciens* strain GV3101 by electroporation and subsequently transformed into wild-type *Arabidopsis thaliana* (Col-0) using the floral dip method [34]. Transgenic T1 plants were selected on MS medium containing hygromycin, and homozygous T3 lines were obtained for functional analysis.

### Identification of *Arabidopsis thaliana* mutants in *AtNPF6.3/AtNRT1.1*

The *atnpf6.3/atnrt1.1* T-DNA insertion mutant (SALK_097431C) and wild-type *Arabidopsis thaliana* (Col-0) were obtained from the *Arabidopsis* Biological Resource Center (ABRC). Homozygous mutants were identified by PCR-based screening. Total genomic DNA was extracted from leaves of 3-week-old soil-grown plants using the hexadecyl trimethyl ammonium bromide (CTAB) method. PCR was performed with gene-specific primers (LP/RP) and a T-DNA border primer (BP) (Supplementary Table S9). Each 25 µL PCR reaction contained 2 µL genomic DNA, 1 µL of each primer (10 µM), 12.5 µL 2× Taq PCR Master Mix (Vazyme, Nanjing, China), and ddH₂O to 25 µL. The PCR program was as follows: Initial denaturation at 95 °C for 5 min, 36 cycles of 95 °C for 30 s, 58 °C for 30 s, and 72 °C for 40 s, followed by a final extension at 72 °C for 5 min. PCR products were resolved on a 1.0% agarose gel to distinguish homozygous mutants from the wild-type plants. To confirm the knockout of *AtNPF6.3/AtNRT1.1*, total RNA was extracted from rosette leaves of homozygous mutants and wild-type plants using the TaKaRa MiniBEST Plant RNA Extraction Kit. First-strand cDNA was synthesized from 1 µg of DNase I-treated RNA using the PrimeScript RT reagent kit. The absence of full-length *AtNPF6.3/AtNRT1.1* transcript in the mutant was verified by qRT-PCR using gene-specific primers.

### Phenotype analysis

To functionally characterize *GmNPF6.8*, T3 homozygous overexpression seeds, *atnpf6.3/atnrt1.1* mutants, and WT plants were surface-sterilized with 12% sodium hypochlorite for 15 min, followed by 8–10 rinses with sterile distilled water. The seeds were then placed in culture dishes and vertically grown in a growth incubator at 25 °C under a 16 h light/8 h dark photoperiod for 7 days to ensure uniform germination and seedling growth. For the nitrogen response assay, 7-day-old seedlings of similar size were carefully transferred to fresh MS medium supplemented with either 2.5 mM normal nitrogen (NN) or 0.25 mM low nitrogen (LN). Seedlings were vertically cultured for another 7 days under the same conditions. Primary root length and lateral root density were recorded using a digital camera, and root traits were quantified with ImageJ software. Each treatment included at least four seedlings per genotype, and all assays were performed independently in triplicate.

### Transformation of *GmNPF6.8* transgenic soybean hairy roots

The pHZM33-35 S:*GmNPF6.8* vector was successfully constructed and introduced into *Agrobacterium tumefaciens* strain K599. The transformation of soybean hairy roots followed the method described by previous studies [35]. Seeds were disinfected with chlorine gas and germinated on 1/2MS medium at 25 °C for 5–6 days under a 16 h light/8 h dark photoperiod. Cotyledons were wounded on the backside with a blade and then immersed in a culture of *A. rhizobium* K599 carrying the construct. The seedings were placed upside down on MS medium. To suppress bacterial overgrowth, 250 µg/mL carbenicillin and cefotetan were added to the medium, and plates were incubated in the dark at 25 ℃. Putative transformed hairy roots, approximately 18–20 days after emergence and 4–6 cm in length, were used for further experiments. For quantitative analysis of root architecture, hairy root systems were carefully washed, and high-resolution images were captured using a flatbed scanner. Root traits were analyzed with WinRHIZO Pro 2016a, including total root length, number of root tips, root surface area, root volume, and average root diameter. Each transgenic line and the wild-type control included at least three independent biological replicates.

### Dual luciferase assays

The full-length coding sequence of *GmARF11* was cloned into pGreenII 62-SK to generate the effector vector pGreenII 62-*GmARF11*-SK. The promoter region of *GmNPF6.8* was cloned into the reporter vector pGreenII 0800-LUC to generate pGreenII 0800-*GmNPF6.8*-LUC. Both constructs were independently introduced into *Agrobacterium tumefaciens* strain GV3101 by chemical transformation. Single colonies carrying the respective plasmids were cultured in 10 mL of lysogeny broth (LB) liquid medium with appropriate antibiotics, and shaken at 200 rpm for 16–24 h at 28 ℃ until the OD600 reached 0.8. For co-infiltration, 5 mL of pGreenII 62-*GmARF11*-SK culture was mixed with 5 mL of pGreenII 0800-*GmNPF6.8*-LUC culture in a 15 mL microcentrifuge tube. The bacterial pellet was resuspended in 10 mL of osmotic buffer (10 mM MES, pH 5.6; 10 mM MgCl₂; 100 µM acetosyringone) and incubated at room temperature for 2–3 h without shaking. Healthy 5-week-old *Nicotiana benthamiana* plants were used for infiltration. A needle-free syringe was used to infiltrate the bacterial suspension into the abaxial surface of fully expanded leaves. Infiltrated plants were kept in the dark for 24 h and then transferred to a growth chamber under normal light/dark cycles for an additional 48 h. Luciferase activity was measured using the Dual-Luciferase Reporter Assay Kit (Vazyme, Nanjing, China).

### Statistical analysis

Statistical analyses were performed with Student’s *t-*test and one-way analysis of variance using the statistical software Statistical Product and Service Solutions (SPSS) 17.0. The significance criteria used were *P < 0.05 and **P < 0.01, respectively.

## Results

### Identification of *NPF* genes in soybean

In this study, a total of 126 *GmNPF* genes were identified in the Wm82.a4.v1 genome using BLASTP with 53 *Arabidopsis thaliana* NPF proteins as queries and by screening for the presence of the PTR2 domain (Supplementary Table S1). Compared with a previous study [6], 7 new *GmNPF* genes were identified, while 15 genes were excluded because they were either misannotated in the earlier genome version or lacked the PTR2 domain. The newly identified genes were named according to their subfamily classification, which was determined by phylogenetic analysis of all NPF proteins from soybean and *Arabidopsis* [6].

### Sequence characteristics and chromosomal locations of soybean *NPF* genes

The basic characteristics of the GmNPF family members are summarized in Supplementary Table S1. The proteins showed considerable diversity in size and charge. *GmNPF2.13* is the smallest protein (115 amino acids), whereas *GmNPF6.3* is the largest (631 amino acids). Molecular weights ranged from 12.67 to 71.00 kDa, and theoretical isoelectric points (pI) varied from 5.19 (*GmNPF1.11*, acidic) to 10.04 (*GmNPF5.45*, basic). Most proteins were predicted to be hydrophobic, with *GmNPF7.7* as an exception, exhibiting the lowest aliphatic index (68.55) and a negative GRAVY value (-0.327), indicating a hydrophilic character. This broad variation in physicochemical properties suggests potential functional diversification among *GmNPF* genes.

**Fig. 1:**
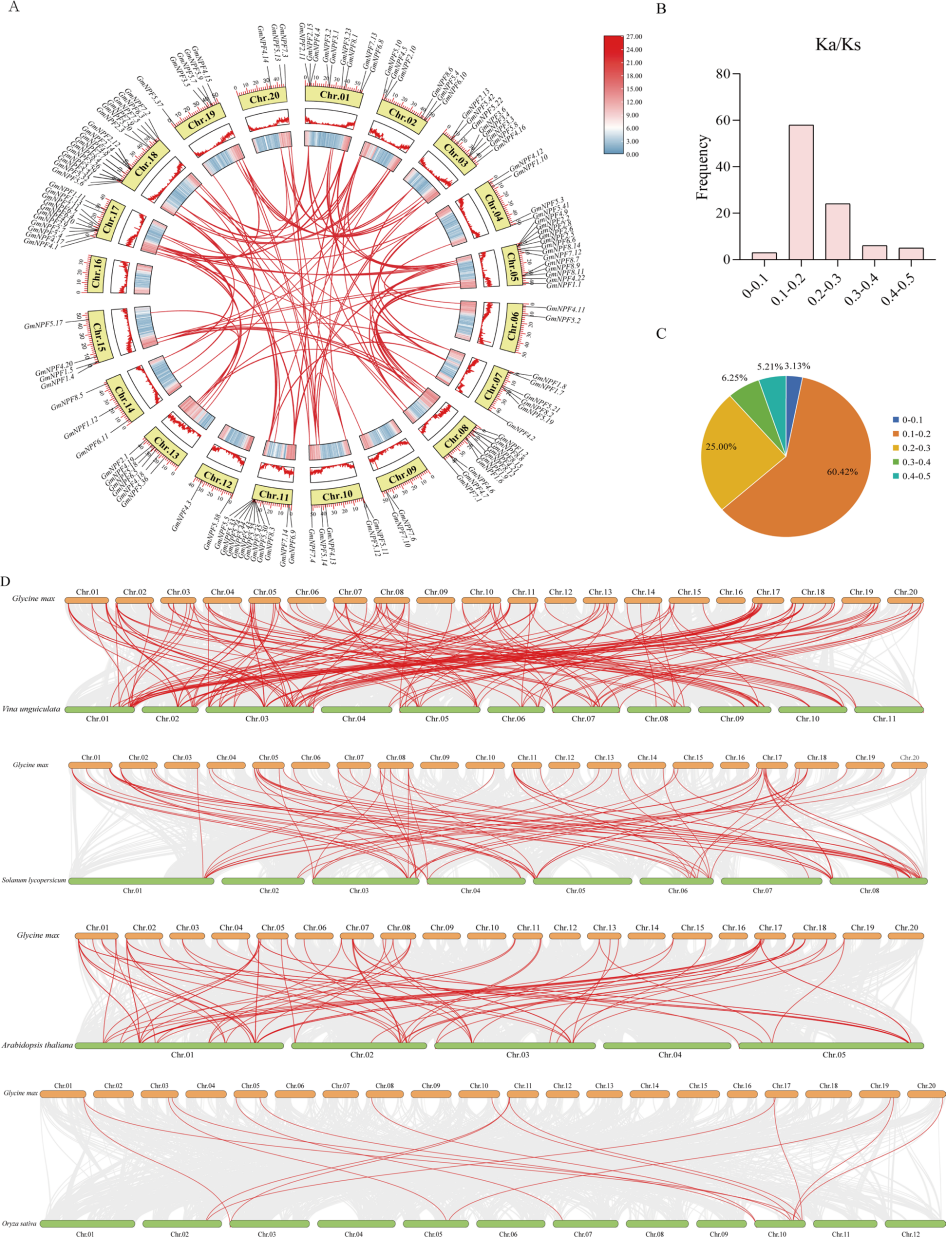
Chromosomal distribution of *GmNPF* genes and frequency distribution of Ka/Ks values for duplicated *GmNPF* gene pairs. **A** Chromosomal relationships of the *GmNPF* gene in the soybean genome. Segmentally duplicated gene pairs are connected by red lines. The gene density of 100-kb genetic interval on each chromosome is represented by a heatmap. **B** Frequency distribution of Ka/Ks values of duplicated *GmNPF* gene pairs. **C** The proportion of Ka/Ks value occupancy in different distributions of *GmNPF* gene pairs. **D** Synteny analysis of the *NPF* genes between soybean and four representative species. Collinear regions within the genomes are connected by gray lines, while syntenic *NPF* gene pairs are highlighted with red lines

Chromosomal positions of the *GmNPF* genes were determined based on their physical locations in the soybean genome. Gene density per chromosome was calculated in 100-kb intervals (Supplementary Table S2) and visualized as a color gradient, with blue representing low density and red high density. The 126 *GmNPF* genes were unevenly distributed across 19 chromosomes, with 2 to 16 genes per chromosome. Chromosome 18 contained the highest number of genes (16), followed by chromosome 5 (13 genes), while chromosome 6, 9, and 12 each contained only 2 genes (Fig. 1A). This distribution indicates that *GmNPF* genes are unevenly arranged across the soybean genome.

### Collinearity analysis of *GmNPF* gene members

Tandem and segmental duplications are major mechanisms leading to the expansion of gene families [36]. To investigate the expansion of the soybean *NPF* gene family, intragenomic collinearity analysis of the 126 *GmNPF* genes was performed using MCScanX. The analysis identified 98 segmental duplication events involving 91 *GmNPF* genes, indicating that segmental duplication is the primary mechanism behind the expansion of this family (Fig. 1A and Supplementary Table S3). The evolutionary pressure on these duplicated genes was assessed using Ka/Ks ratios. Over 85% of the duplicated gene pairs showed ratios below 0.3 (ranging from 0.07 to 0.48), indicating strong purifying selection (Fig. 1B-C and Supplementary Table S4). These results suggest that the *GmNPF* gene family has predominantly evolved under purifying selection.

Interspecific collinearity analysis revealed distinct evolutionary patterns of the *NPF* genes between soybean and other species (Fig. 1D and Supplementary Table S5). A total of 90 *GmNPF* genes showed syntenic relationships with orthologs in *Arabidopsis thaliana* (28), *Oryza sativa* (8), *Solanum lycopersicum* (25), and *Vigna unguiculata* (49), forming 110 cross-species syntenic pairs. These results indicate that the NPF transporter family is more evolutionarily conserved among dicotyledonous species.

### Cis-regulatory element analyses of soybean *GmNPF* genes

To investigate potential regulatory mechanisms underlying the functional diversity of the *GmNPF* genes, cis-regulatory elements in the 2,000 bp upstream promoter regions were analyzed using PlantCARE. The promoters contained numerous stress- and hormone-responsive elements (Fig. S1). For example, *GmNPF5.37*, *GmNPF2.8*, *GmNPF5.36*, and *GmNPF4.21* each harbored more than 7 ABA-responsive elements, while 17 *GmNPF* genes contained more than 6 MeJA-responsive elements in their promoters, suggesting that these genes may be transcriptionally induced by ABA and MeJA (Fig. S2 and Supplementary Table S6). Notably, the *GmNPF4.21* promoter contains 7 ABA-responsive, 5 light-responsive, and 8 MeJA-responsive elements, indicating its potential role in integrating hormonal and environmental signals.

### Analysis of tissue-specific expression patterns of *GmNPF* genes

To investigate the expression profiles of *GmNPF* genes across different tissues and developmental stages, this study analyzed the expression levels of *GmNPF* genes in young leaves, flowers, 1-cm pod, pod shell 10 days after flowering (DAF), pod shell 14 DAF, seed 10 DAF, seed 14 DAF, seed 21 DAF, seed 25 DAF, seed 28 DAF, seed 35 DAF, seed 42 DAF, root and nodule [28]. Rather than simply presenting a complex heatmap of all 112 detected genes (Fig. S3 and Supplementary Table S7), the results systematically identified and categorized genes with distinct expression patterns. This analysis identified both tissue-specific and constitutively expressed *GmNPF* members (Supplementary Table S10). Specifically, *GmNPF5.13*, *GmNPF5.14*, and *GmNPF5.38* were constitutively highly expressed, suggesting they act as housekeeping genes during soybean development. *GmNPF1.2*, *GmNPF5.2*, *GmNPF5.25*, *GmNPF5.3*, *GmNPF5.29*, *GmNPF5.30*, and *GmNPF8.6* showed high expression in root nodules, indicating potential roles in nodule development. Importantly, *GmNPF6.8* and *GmNPF6.9* displayed strong root-specific expression, suggesting they may participate in root-related processes during soybean development.

### Analysis of expression patterns of *GmNPF* genes under different nitrogen conditions

**Fig. 2:**
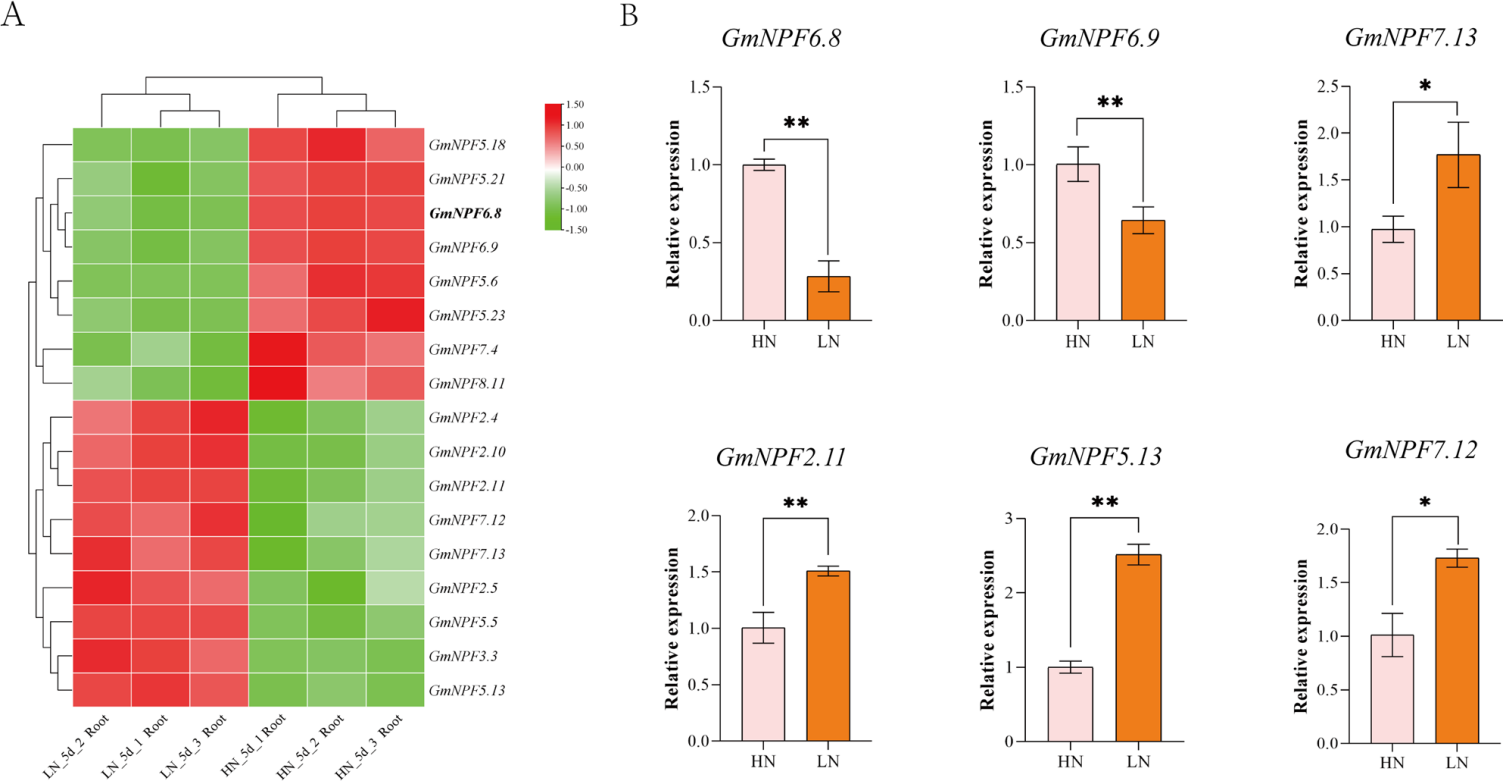
Expression patterns of *GmNPF* genes under different nitrogen treatment. **A** Heat map analysis of genes with significant differential expression in nitrogen response. The scale bar represents the Log_2_ standardized expression. **B** Relative expression patterns of 6 *GmNPF* genes in response to HN and LN treatments. Data are presented as mean ± SD (*n* = 3). Significant differences between HN and LN treatments for each gene are indicated by asterisks (*P < 0.05, **P < 0.01)

To examine the response of *GmNPF* genes to nitrogen stress, RNA-seq data from soybean roots under different nitrogen treatments were analyzed (PPRD). Expression profiles of all 118 detected genes are shown in Fig. S4, with the detailed data provided in Supplementary Table S8. From this analysis, the most significantly differentially expressed genes (DEGs) were highlighted in Fig. 2A and Supplementary Table S12, identifying the core nitrogen-responsive candidates. Nine genes (*GmNPF2.4*, *GmNPF2.10*, *GmNPF2.5*, *GmNPF3.3*, *GmNPF5.13*, *GmNPF5.5*, *GmNPF7.13*, *GmNPF7.12*, and *GmNPF2.11*) were significantly upregulated (*p* < 0.05), whereas *GmNPF6.8* and *GmNPF6.9* were significantly downregulated in roots under low nitrogen stress compared with controls (*p* < 0.01; Fig. 2A). These results suggest that the upregulated genes may promote nitrogen uptake or utilization, while *GmNPF6.8* and *GmNPF6.9* could act as negative regulators of nitrogen stress responses, making them promising candidates for further study.

These findings were validated by qRT-PCR, which strongly confirmed the RNA-seq data (Fig. 2B). Based on these results, *GmNPF6.8* was selected for in-depth functional characterization due to its significant downregulation, root-specific expression, and evolutionary conservation with *AtNPF6.3/AtNRT1.1*. Similarly, the significantly upregulated genes *GmNPF2.11*, *GmNPF7.12*, *GmNPF7.13*, and *GmNPF5.13* were selected for population genetics analysis. These core candidates thus established a molecular foundation for subsequent functional investigations.

### Evolutionary and conservative motif analysis of *GmNPF6.8* gene

To clarify the evolutionary relationships of *GmNPF6.8*, a phylogenetic analysis was conducted using 126 *GmNPF* genes alongside 53 *Arabidopsis thaliana* NPF proteins. The analysis classified the *GmNPF* family into 8 subfamilies, consistent with the *Arabidopsis* NPF family, reflecting evolutionary conservation across dicots (Fig. S5 and Fig S6). Notably, the *GmNPF6* subfamily is evolutionarily conserved across plant species and is known to include core nitrate transporters that integrate nitrate signaling with root development. High bootstrap support (99.51%) and substantial sequence identity (61.34%) indicate that *GmNPF6.8* and *AtNPF6.3/AtNRT1.1* are highly homologous (Fig. S7), with the latter established as a key regulator of nitrate uptake and root development [14]. These results strongly suggest that *GmNPF6.8* may have similar nitrate transport and root regulatory functions in soybean.

Analysis of conserved motifs showed that *GmNPF6.8* contains all 10 conserved motifs of the GmNPF family, including motif 4, a critical component of the PTR2 domain essential for nitrate transport (Fig. S8A-B; Supplementary Table S11 and Fig. S9). The PTR2 domain of *GmNPF6.8* shares 88% sequence identity with that of *AtNPF6.3/AtNRT1.1* (Fig. S7), confirming its structural capacity for nitrate translocation. In contrast, other NPF family members, such as *GmNPF5.44*, *GmNPF6.6*, and *GmNPF3.3*, lack motif 4, suggesting they may not function as nitrate transporters and could be involved in the transporting other substrates.

Furthermore, the intron-exon structure of *GmNPF6.8* aligns with the conserved pattern of the NPF6 subfamily, featuring four exons (Fig. S8C). Although the *GmNPF* family overall shows variation in exon number (1–6), this structural consistency supports the classification of *GmNPF6.8* within the NPF6 subfamily and reinforces its potential functional conservation with *AtNPF6.3/AtNRT1.1*.

### Population genetics and selection analysis of key nitrogen-responsive *GmNPF* genes

**Fig. 3:**
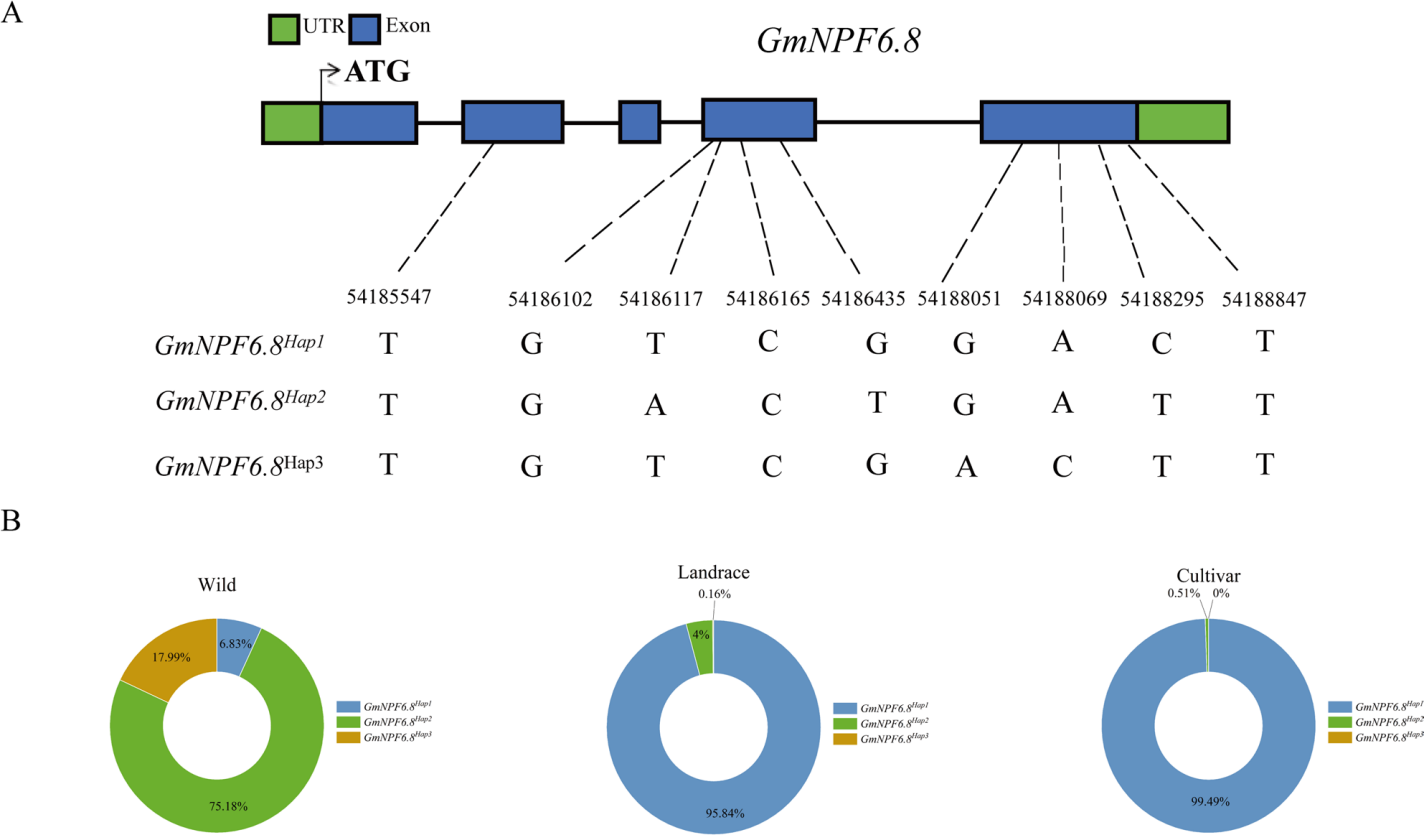
Natural variation and evolutionary analysis of *GmNPF6.8* gene in soybean. **A** Identification of three major haplotypes (Hap1, Hap2, Hap3) within the coding region of *GmNPF6.8*, defined by nine polymorphic sites. UTRs and exons are indicated by green and blue boxes, respectively. **B** Evolutionary analysis of three haplotypes in the coding region of *GmNPF6.8* gene

To identify signatures of artificial selection in key nitrogen-responsive candidates, this study analyzed nucleotide variation in its coding region using the SoyMD database (https://yanglab.hzau.edu.cn/SoyMD/#/variation), a public resource for soybean multi-omics data. Based on resequencing data from 4,068 soybean accessions, 9 single nucleotide polymorphism (SNP) loci in the coding region of *GmNPF6.8* were identified, defining 3 distinct haplotypes (*GmNPF6.8*^*Hap1*^, *GmNPF6.8*^*Hap2*^, *GmNPF6.8*^*Hap3*^). Among the haplotypes, the SNP mutations in *GmNPF6.8*^*Hap1*^ and *GmNPF6.8*^*Hap2*^ were synonymous mutations and did not alter the protein sequence of *GmNPF6.8*, whereas *GmNPF6.8*^*Hap1*^ and *GmNPF6.8*^*Hap3*^ contained 2 nonsynonymous SNPs (SNP 54,188,051 and SNP 54,188,069), which were located outside the core PTR2 domain essential for nitrate transport (Fig. 3A and Supplementary Table S13). Notably, the frequency of *GmNPF6.8*^*Hap1*^ increased dramatically from 6.83% in wild soybeans to 95.84% in landraces and 99.49% in cultivars (Fig. 3B), indicating strong artificial selection during domestication and breeding. This analysis also highlighted a clear distinction between the paralogs *GmNPF6.8* and *GmNPF6.9*. Although *GmNPF6.9* displayed a similar expression pattern to *GmNPF6.8* under low nitrogen conditions, its haplotype frequency increased only modestly from 76.47% in wild soybeans to 88.70% in cultivars (Fig. S10), suggesting it was not a major target of selection. Similarly, *GmNPF2.11* and *GmNPF5.13* underwent strong artificial selection, with the frequencies of their key haplotypes (*GmNPF2.11*^*Hap1*^ and *GmNPF5.13*^*Hap2*^) rising substantially from 8.39% to 81.21% and 8.45% to 35.31% (Fig. S11 and Fig. S12). In contrast, the frequency of *GmNPF7.13*^*Hap1*^ increased only from 87.85% in wild soybeans to 96.19% in selected varieties, a total rise of 8.34%, representing the smallest increase (Fig. S13). No SNPs were detected in the coding region of *GmNPF7.12*, indicating that this gene was not subjected to strong artificial selection. Overall, these population genetics findings reveal a series of selection pressures between nitrogen responsive *GmNPF* genes. *GmNPF6.8* gene exhibits significant selection pressure, and its key haplotype is almost completely fixed in breeding varieties. At the same time, this gene is significantly downregulated under low nitrogen conditions, indicating that it is a key regulatory factor for root structure adaptation to nitrogen regulation.

### Subcellular localization of the GmNPF6.8 protein and spatial expression patterns

**Fig. 4:**
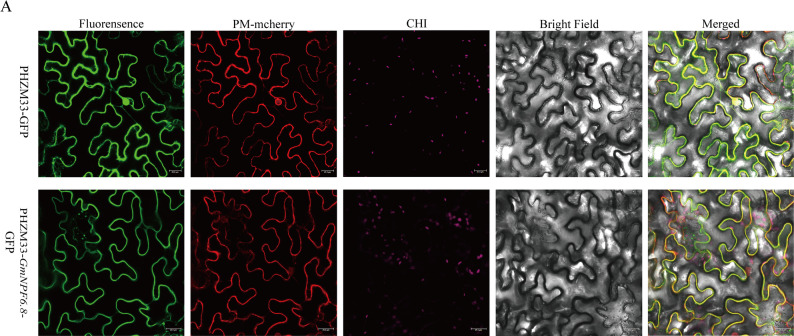
Subcellular localization and spatial expression patterns of *GmNPF6.8* in soybean. **A** Subcellular localization of the *GmNPF6.8*-GFP fusion protein in *Nicotiana benthamiana* leaves. The plasma membrane was marked by co-expression with *AtLTI6B*-mCherry. The empty pHZM33-GFP vector served as a negative control. Experiments were independently repeated three times with consistent results. Scale bar = 25 μm. CHI: Chlorophyll autofluorescence

To further investigate the function of *GmNPF* genes in soybean, *GmNPF6.8*, a homolog of *AtNPF6.3/AtNRT1.1* [37], was selected for further functional characterization. For subcellular localization, the *GmNPF6.8*-GFP fusion construct was co-expressed with the plasma membrane marker *AtLTI6B*-mCherry in *Nicotiana benthamiana* leaf epidermal cells. Green fluorescence from *GmNPF6.8*-GFP was observed on the plasma membrane, overlapping with the red fluorescence of *AtLTI6B*-mCherry, indicating that GmNPF6.8 is primarily localized to the plasma membrane (Fig. 4). This localization is consistent with its predicted role as a nitrate transporter. Furthermore, root-specific expression of *GmNPF6.8* was confirmed by qRT-PCR (Fig. S14), in agreement with the RNA-seq data (Fig. S3). Collectively, these results confirm that *GmNPF6.8* functions as a key mediator of nitrate-related processes at the plasma membrane of root cells.

### *GmNPF6.8* overexpression inhibits root development in *Arabidopsis* under NN and LN conditions

**Fig. 5:**
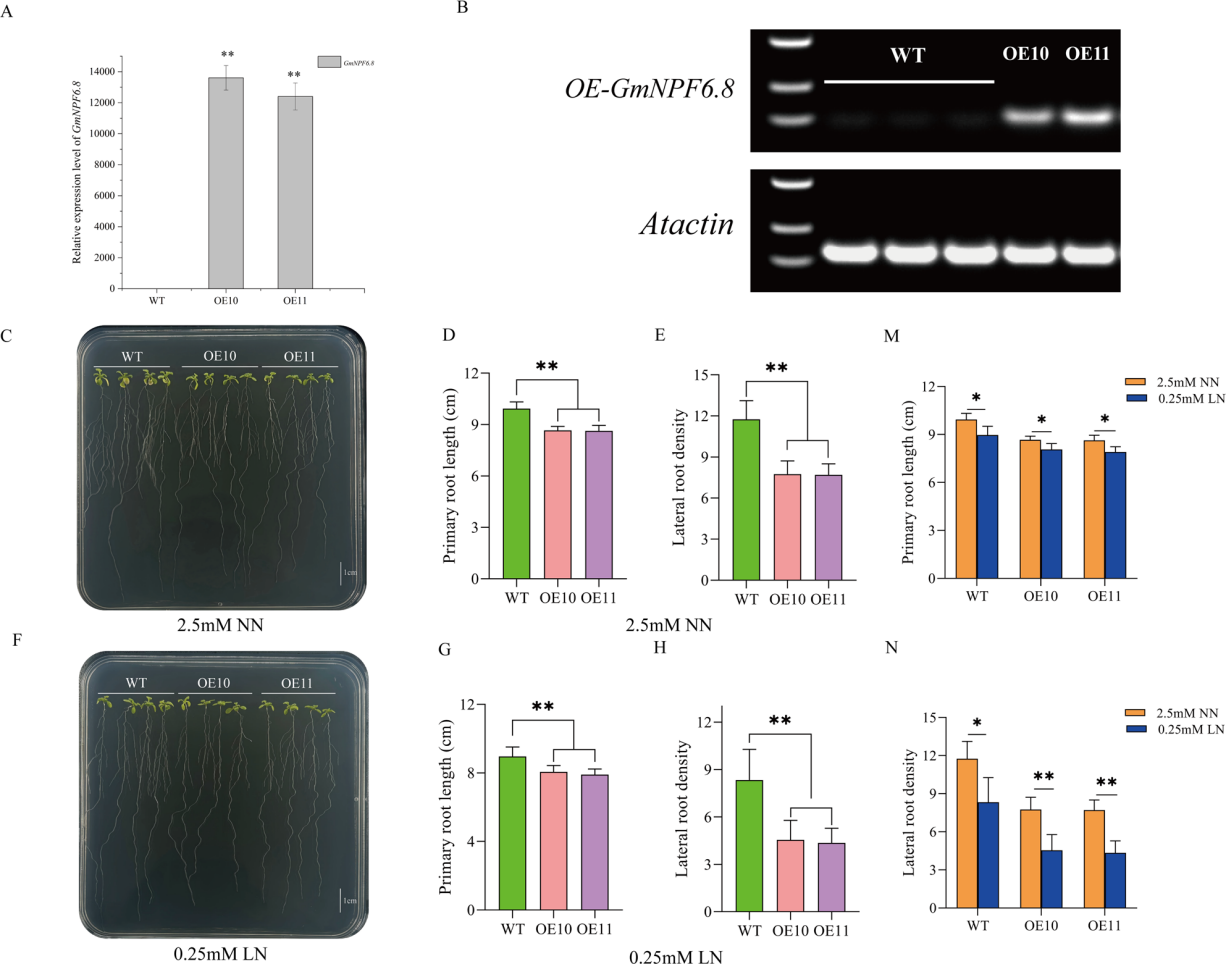
Overexpression of *GmNPF6.8* alters root architecture in *Arabidopsis* under different nitrogen conditions. **A** qRT-PCR analysis of *GmNPF6.8* expression levels in WT and two independent overexpression transgenic lines (OE10, OE11). **B** The RT-PCR verification of *GmNPF6.8* transcript levels in OE10, OE11, and WT. **C** Root phenotypes of WT, OE10, and OE11 grown on MS medium supplemented with 2.5mM NN. Scale bar = 1 cm. **D**,** E** Primary root length (**D**) and Lateral root density (**E**) under NN conditions. **F** Root phenotypes of WT, OE10, and OE11 grown on MS medium supplemented with 0.25mM LN. Scale bar = 1 cm. **G**,** H** Primary root length (**G**) and Lateral root density (**H**) under LN conditions. **M** Comparison of primary root length (**M**) and lateral root density (**N**) between NN and LN conditions. Data are presented as mean ± SD (*n* = 12 plants per genotype from three biological replicates, each containing four plants). Asterisks indicate significant differences compared with WT (*P < 0.05, **P < 0.01)

To further investigate the biological function of *GmNPF6.8*, we generated *Arabidopsis* overexpression lines (OE10 and OE11) and assessed their root development under different nitrogen conditions. qRT-PCR and genomic PCR confirmed the successful integration and high expression of *GmNPF6.8* in these lines (Fig. 5A-B). Phenotypic analysis revealed that under normal nitrogen (NN) conditions, the overexpression lines exhibited significantly shorter primary roots and lower lateral root density compared with wild-type plants (Fig. 5C-E). Under low nitrogen (LN) conditions, these root development defects were even more pronounced, with both primary root length and lateral root density markedly reduced relative to the wild type (Fig. 5F-H). Integrated analysis of root traits under different nitrogen levels confirmed that, regardless of NN or LN conditions, the primary root length and lateral root density of OE10 and OE11 lines were consistently and significantly lower than those of wild-type plants (*P* < 0.05; Fig. 5M-N). Notably, under LN treatment, the overexpression lines did not show adaptive root system enhancement; instead, their lateral root density decreased further compared with NN conditions (*P* < 0.05; Fig. 5N). In summary, overexpression of *GmNPF6.8* markedly inhibits primary root growth and lateral root formation in *Arabidopsis*, reducing root plasticity under low nitrogen. These results suggest that *GmNPF6.8* functions as a negative regulator of root development, potentially acting within the nitrogen signaling network that modulates root architecture.

### Loss of function of *AtNPF6.3/AtNRT1.1* significantly promoted root development across nitrogen conditions

**Fig. 6:**
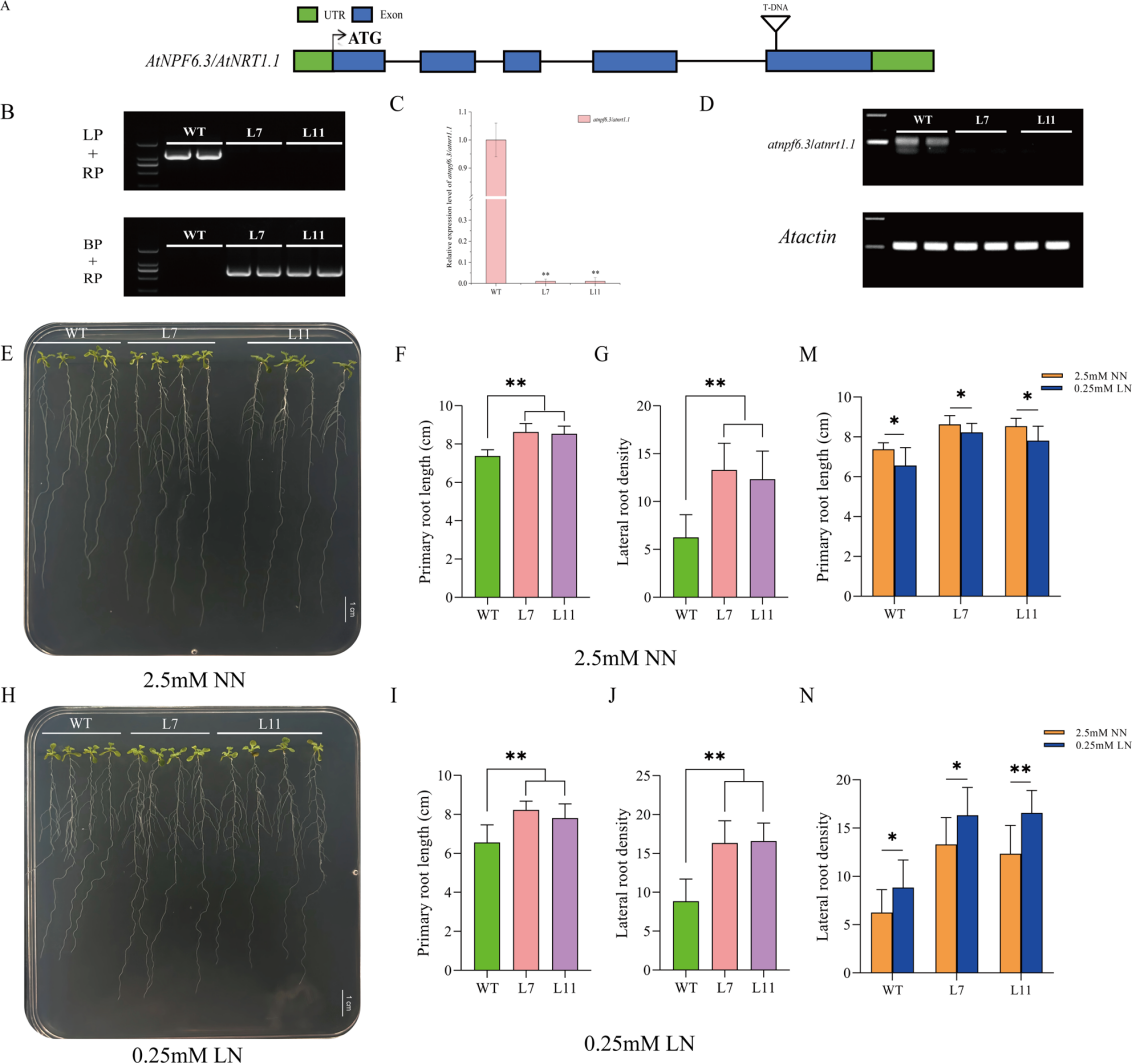
Loss of function of *AtNPF6.3/AtNRT1.1* promotes root development in *Arabidopsis* under different nitrogen conditions. **A** Gene structure of *AtNPF6.3/AtNRT1.1* showing the T-DNA insertion sites within exons. Green boxes represent UTRs; blue boxes represent exons. **B** Identification of *atnpf6.3/atnrt1.1* mutant homozygous transgenic lines. **C** Relative expression levels of *AtNPF6.3/AtNRT1.1* in WT and homozygous mutant lines. **D** RT-PCR verification of *AtNPF6.3/AtNRT1.1* transcript levels in homozygous mutant lines. **E** Root phenotype of WT and *atnpf6.3/atnrt1.1* mutants grown on MS medium with 2.5mM NN. Scale bar = 1 cm. **F**,** G** Primary root length (**F**) and Lateral root density (**G**) under NN conditions. **H** Root phenotype of WT and *atnpf6.3/atnrt1.1* mutants grown on MS medium with 0.25mM LN. Scale bar = 1 cm. **I**,** J** Primary root length (**I**) and Lateral root density (**J**) under LN conditions. **M** Comparison of primary root length (**M**) and lateral root density (**N**) between NN and LN conditions. Data are presented as mean ± SD (*n* = 12 plants per genotype from three biological replicates, each containing four plants). Asterisks indicate significant differences compared with WT (*P < 0.05, **P < 0.01)

Given the evolutionary relationship between *GmNPF6.8* and *AtNPF6.3/AtNRT1.1* (Fig. S5), this study examined whether the function of *GmNPF6.8* in nitrogen-responsive root development is conserved. Two homozygous T-DNA insertion mutants of *atnpf6.3/atnrt1.1* (L7 and L11) were analyzed. Genotyping and qRT-PCR confirmed that both lines were loss-of-function mutants (Fig. 6A-D). Phenotypic analysis showed that under 2.5 mM NN conditions, the primary root length and lateral root density of L7 and L11 were significantly higher than those of wild-type plants (*p* < 0.01; Fig. 6E-G). Under 0.25 mM LN conditions, this enhanced root growth phenotype persisted, with the mutants still displaying significantly greater primary root length and lateral root density compared with WT (*p* < 0.01; Fig. 6H-J). Further analysis across different nitrogen levels indicated that, regardless of nitrogen supply, L7 and L11 consistently exhibited longer primary roots and higher lateral root density than WT (*p* < 0.05; Fig. 6M, N). Notably, low nitrogen treatment further increased lateral root density in the mutants compared to NN conditions (*p* < 0.05; Fig. 6N). In summary, loss of *AtNPF6.3/AtNRT1.1* function markedly promotes root development in *Arabidopsis*, strongly supporting the functional conservation of *GmNPF6.8* and *AtNPF6.3/AtNRT1.1* as negative regulators of root growth.

### Overexpression of the *GmNPF6.8* gene negatively regulates root development

**Fig. 7:**
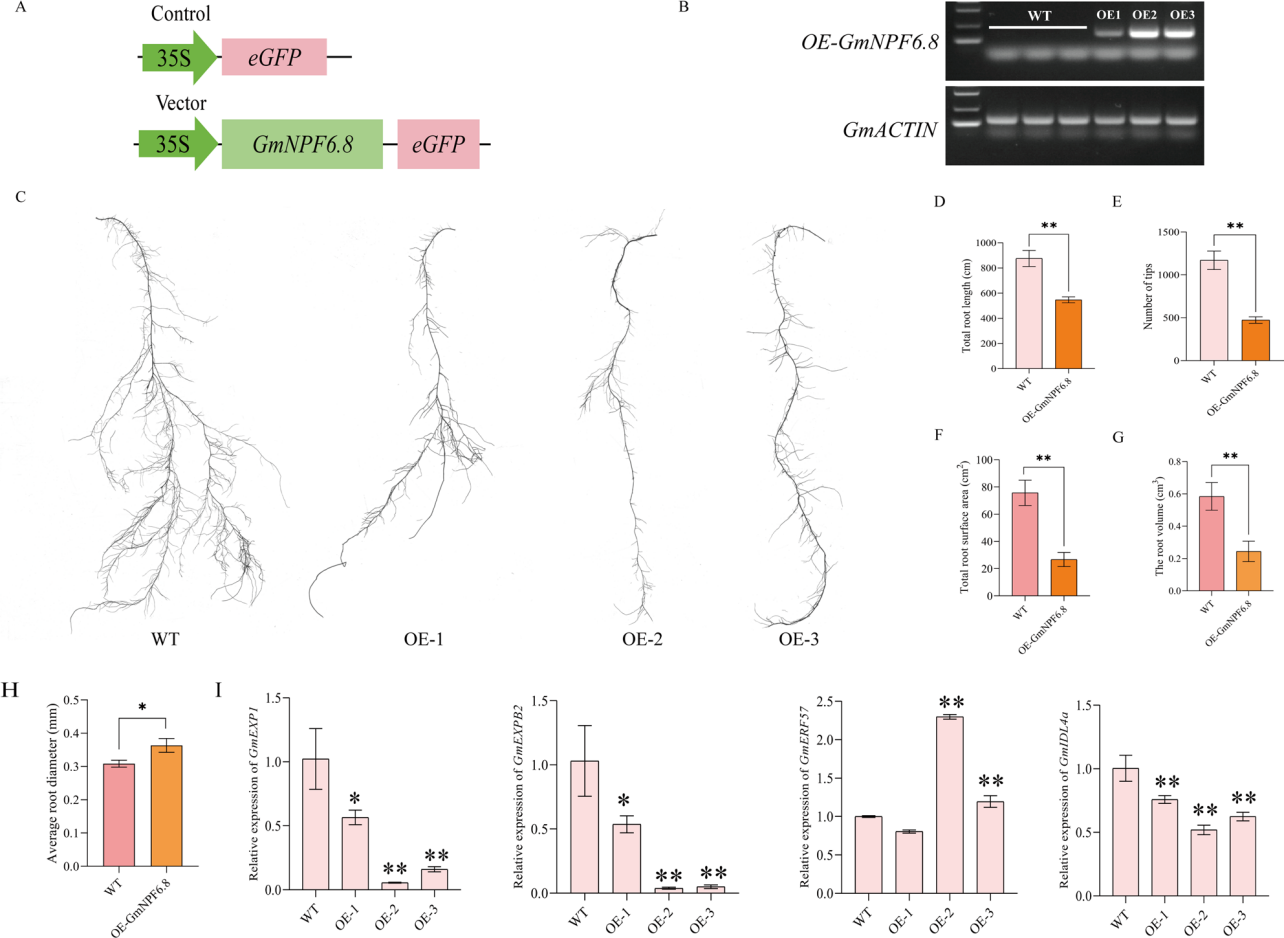
Overexpression of *GmNPF6.8* modulates root architecture in transgenic soybean hairy roots. **A** Schematic of the *GmNPF6.8* overexpression vector. **B** RT-PCR analysis of *GmNPF6.8* transcript levels in WT and three independent transgenic lines. **C** Representative root phenotypes of WT and *GmNPF6.8*-overexpression hairy-root transgenic plants. **D**,** E**,** F**,** G**,** H** Quantification of total root length (**D**), Number of root tips (**E**), Total root surface area (**F**), The root volume (**G**), and Average root diameter (**H**). **I** Relative expression levels of key genes associated with root architecture in WT and *GmNPF6.8*-OE lines. For panels D-I, data are presented as mean ± SD (*n* = 3 independent biological replicates). Asterisks indicate significant differences compared with WT (*P < 0.05, **P < 0.01)

To investigate the biological function of *GmNPF6.8* in soybean root development, transgenic soybean hairy roots overexpressing the gene were generated (Fig. 7A). Three independent lines (OE-1, OE-2, OE-3) with high transcript levels were selected for further analysis (Fig. 7B). Phenotypic assessment showed that overexpression of *GmNPF6.8* significantly inhibited root growth, resulting in shorter total root length, fewer root tips, and markedly reduced total root surface area and root volume compared with WT (*p* < 0.05; Fig. 7C-H).

To explore the molecular mechanism of root inhibition mediated by *GmNPF6.8*, the expression of four root development-related genes (*GmEXP1* [38], *GmEXPB2* [39], *GmERF57* [40], and *GmIDL4a* [41]) was analyzed. Compared with WT, transcript levels of *GmEXP1*, *GmEXPB2*, and *GmIDL4a* were significantly downregulated in the overexpressing lines, whereas the transcript level of *GmERF57* expression was significantly upregulated (*p* < 0.05; Fig. 7I). These results indicate that *GmNPF6.8* negatively regulates soybean root development, likely through transcriptional modulation of key root growth-related genes.

### *GmARF11* directly binds to the *GmNPF6.8* promoter and inhibits its transcriptional activity

**Fig. 8:**
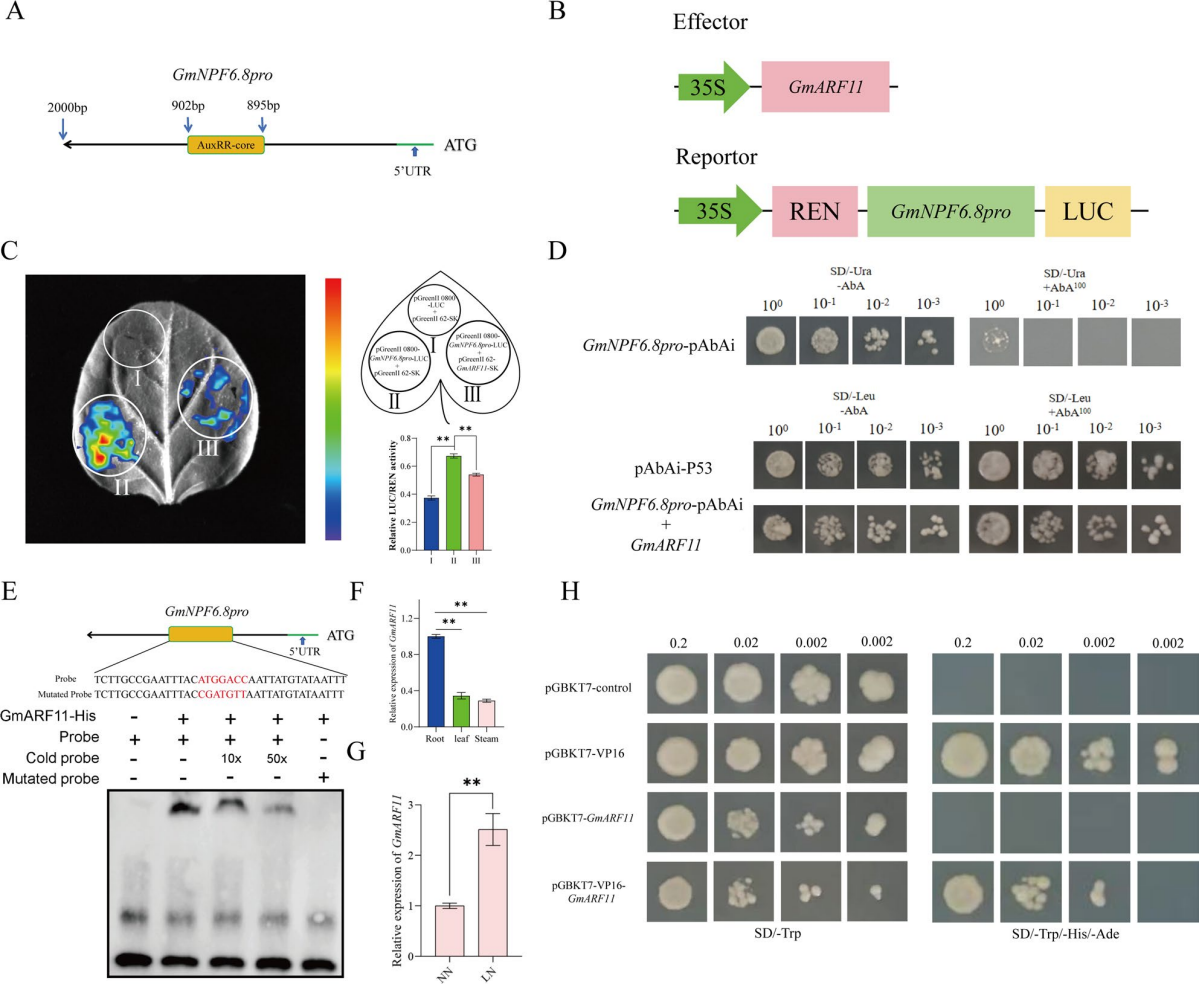
*GmARF11* directly binds to the promoter of *GmNPF6.8* and inhibits its transcriptional activity. **A** Schematic diagram of the *GmNPF6.8* promoter. **B** Experimental design for transient expression analysis using 35 S :: *GmARF11* as the effector and ProGmNPF6.8 :: LUC as the reporter. **C** Effect of *GmARF11* on *GmNPF6.8* promoter activity in *Nicotiana benthamiana* leaves, measured using a LUC-based reporter system. The relative LUC/REN ratio quantifies transcriptional regulation by *GmARF11*. Error bars indicate SD from at least three independent replicates. Asterisks denote significant differences compared with the control (* *P* < 0.05, * * *P* < 0.01). **D** Y1H detection of *GmARF11* and *GmNPF6.8* promoter binding in vitro. 10^− 1^, 10^− 2^, and 10^− 3^ respectively indicate that the yeast concentration has been diluted by 10, 100, and 1000 times. 3-AT represents 3-amino-1,2,4-triazole. **E** EMSA showing that *GmARF11* could directly bind to the AuxRR-core element in the promoter of *GmNPF6.8*. A single probe was used as a negative control in lane 1; recombinant GmARF11-His protein was displayed in lanes 2–5; 10× and 50× unlabeled probes were added as competitors in lanes 3–4, respectively; and a biotin-labeled mutant probe (ATGGACC changed to CGATGTT) was added in lane 5. **F** Relative expression levels of *GmARF11* in roots, stems and leaves. **G** Relative expression patterns of *GmARF11* gene in response to HN and LN treatments. **H** Transcriptional activity analysis of GmARF11 protein. *GmARF11* was cloned and inserted it into pGBKT7 and pGBKT7-VP16 vectors. Transform pGBKT7, pGBKT7-VP16, and recombinant vectors into Y2HGold yeast strains, and culture on SD/-Trp and SD/-Ade/-His/-Trp plates for 3 days

Previous promoter analysis indicated that *GmNPF6.8* contains an AuxRR-core cis element (Fig. S1), a known motif recognized by auxin response factors (ARFs) [42, 43]. Given that *GmARF11* shares the closest homology with tobacco *NtARF11* (Fig. S15), it was hypothesized that *GmARF11* may perform a similar regulatory role in soybean. To investigate whether *GmNPF6.8* is transcriptionally regulated by *GmARF11*, a yeast one-hybrid (Y1H) assay was first conducted. The results showed that *GmARF11* directly binds to the *GmNPF6.8* promoter fragment containing the AuxRR-core element (Fig. 8D). To further validate this interaction in planta, a dual luciferase (LUC) reporter assay was performed in tobacco leaves. Compared with the control, co-expression of the *GmARF11* effector with the *GmNPF6.8* promoter-driven LUC reporter significantly repressed LUC activity (Fig. 8B-C), indicating that *GmARF11* functions as a transcriptional repressor of *GmNPF6.8*. Electrophoretic mobility shift assays (EMSA) were subsequently used to confirm specific binding. *GmARF11* bound to probes containing the AuxRR-core motif, and the binding gradually weakened with increasing concentrations of unlabeled competitor probes (Fig. 8E). No binding was observed with mutant probes lacking the AuxRR-core motif (Fig. 8E), confirming that *GmARF11* specifically recognizes this cis-element. To explore the biological relevance of this regulatory interaction, the expression patterns of *GmARF11* and *GmNPF6.8* were analyzed in soybean roots under LN stress. *GmARF11* was highly expressed in roots (Fig. 8F), and its expression was significantly induced by LN treatment (Fig. 8G). This gene has transcriptional inhibitory activity (Fig. 8H), which is negatively correlated with the downregulation of *GmNPF6.8* (Fig. 2B). Overall, these results indicate that *GmARF11* directly binds to the *GmNPF6.8* promoter and represses its transcription, providing a potential mechanism for the LN-induced downregulation of *GmNPF6.8*.

## Discussion

NPF genes play essential roles in nitrate uptake and transport, processes critical for plant growth and development. The NPF gene family has been identified and characterized in multiple plant species, including *Arabidopsis thaliana* [7], *Oryza sativa* [8], *Triticum aestivum* [9], *Zea mays* L. [10, 11], *Brassica napus* L. [12], and *Gossypium* spp [13]. However, a comprehensive analysis of the NPF family in soybean, an important legume crop, has been lacking. In this study, we performed a genome-wide analysis of the *GmNPF* gene family, examining their phylogenetic relationships, chromosomal distribution, gene structures, conserved motifs, synteny, and expression patterns. In addition, we functionally characterized *GmNPF6.8*, a homolog of *AtNPF6.3/AtNRT1.1*, to investigate its role in root development and response to low nitrogen stress.

A total of 126 *GmNPF* genes were identified in the Wm82.a4.v1 genome, a number differing from a previous reports based on older assemblies [6]. These genes were unevenly distributed across 19 chromosomes (Fig. 1A) and classified into 8 subfamilies (Fig. S5), consistent with patterns observed in *Arabidopsis* [7], *Nicotiana tabacum* [44], *Zea mays* [10, 11], and *Triticum aestivum* [9]. The *GmNPF5* subfamily was the largest, containing 36 members, reflecting the prevalence of NPF5 as the largest subfamily in other species. As an ancient polyploid with a highly repetitive genome, approximately 75% of soybean genes exist in multiple copies [45]. The results revealed that segmental duplication has been the primary mechanism driving *GmNPF* family expansion (Fig. 1A and Supplementary Table S3), a mechanism commonly observed in plant gene family evolution [36, 46]. Furthermore, Ka/Ks analysis indicated that most *GmNPF* gene pairs have undergone strong purifying selection (Fig. 1B-C), suggesting that their functions are highly conserved, consistent with observations in *cotton* and *Setaria* [13, 47].

Cis-regulatory elements act as key molecular switches that control dynamic gene expression networks, regulating diverse biological processes, such as responses to abiotic stress, hormone signaling, and development [48]. Promoter analysis revealed a high abundance of stress- and hormone-related cis-regulatory elements in *GmNPF* genes (Fig. S1; Fig. S2 and Supplementary Table S6), suggesting their potential roles in mediating soybean responses to environmental cues and hormonal regulation. This inference is supported by functional studies of *NPF* genes in other species. For example, ABRE elements in the promoter region of the *MdNRT1.5/MdNPF7.3* gene are required for ABA-mediated expression [49], providing a precedent for the functional relevance of similar cis-elements in *GmNPF* promoters.

Gene expression patterns across tissues and developmental stages provide critical insights into potential gene function. Tissue-specific expression further highlights the roles of individual genes [10, 50]. In soybean, *GmNPF* genes displayed diverse spatiotemporal expression patterns (Fig. S3 and Supplementary Table 10). Notably, *GmNPF6.8* and *GmNPF6.9* showed root-specific expression, indicating specialized roles in root development. In contrast, several *GmNPF5* genes were highly expressed in nodules, suggesting involvement in symbiotic nitrogen fixation, whereas *GmNPF5.13*, *GmNPF5.14*, and *GmNPF5.38* were constitutively expressed, consistent with housekeeping functions. Tissue-specific expression is a conserved feature across NPF families in plants. For example, *AtNPF2.5* is mainly expressed in root cortex cells and induced by salt stress [23], *SiNPF6.3* is predominantly expressed in leaves of *Setaria italica* [47], *OsNPF4.1*/*OsSP1* is highly expressed in young rice panicles [51], and *GmNPF7.5* is primarily expressed in soybean root vascular tissues and induced in the cortex and epidermis under NaCl treatment [52]. These observations collectively highlight the close relationship between tissue-specific expression and functional specialization in the NPF family.

Nitrogen availability markedly influenced the expression of *GmNPF* genes, highlighting their diverse roles in nitrate transport and adaptation to LN conditions. Under LN stress, *GmNPF5.13*, *GmNPF5.5*, *GmNPF7.13*, *GmNPF7.12*, *GmNPF7.14*, and *GmNPF2.11* were significantly upregulated, suggesting potential positive roles in nitrogen uptake. In contrast, *GmNPF6.8* and *GmNPF6.9* were significantly downregulated (Fig. 2), indicating possible negative regulatory functions in the low-nitrogen response pathway. This expression pattern is consistent with that of *GhNPF6.14* in *cotton* under nitrogen deficiency [13]. Together, these findings reveal the functional diversity and contrasting regulatory roles of *GmNPF* family members in response to changing nitrogen conditions.

A key innovation of this study lies in the integration of population genetics analysis with functional characterization. The haplotype frequency of *GmNPF6.8*^*Hap1*^ increased dramatically from less than 7% in wild soybeans to nearly 99.5% in cultivated varieties (Fig. 3B), indicating that this gene has experienced one of the strongest signals of artificial selection within the soybean nitrate transporter family. In contrast, its paralog *GmNPF6.9* shows only weak selection, further highlighting the unique agronomic importance of *GmNPF6.8*. This strong directional selection is consistent with the research results of *OsNPF5.5* (*OsNRT1.1B*) in rice. The selection characteristics of its indica haplotype reflect the differentiation of nitrogen utilization during rice domestication, and are also related to nitrogen utilization efficiency, root stem nitrate transport capacity, and upregulation of nitrate response genes [53]. Importantly, this study provides the first direct evidence linking nitrate transporter function to the regulation of root architecture during crop domestication.

Functionally, *GmNPF6.8* acts as a negative regulator of root growth, as evidenced by consistent inhibition of root development in both *Arabidopsis* overexpression lines and soybean hairy roots. This contrasts with the positive regulatory roles of *AtNPF6.3*/*AtNRT1.1* and *MdNRT1.1* [54, 55], highlighting functional diversification within the NPF6 subfamily. Conversely, our research results are consistent with the reports of *MtNPF6.5* and *SpNRT1.1* [56, 57], suggesting that nitrate-mediated root inhibition may represent a conserved adaptive strategy under specific nitrogen regimes. The strong artificial selection of a root growth-inhibitory factor may reflect a breeding-driven strategy to optimize resource allocation, thereby enhancing yield under nitrogen-sufficient conditions.

Mechanistically, *GmARF11* expression is induced under LN conditions (Fig. 8G), and directly binds to the AuxRR-core element in the *GmNPF6.8* promoter to repress its transcription (Fig. 8B-E). These results indicate that *GmARF11* mediates the downregulation of *GmNPF6.8* in response to LN. As an auxin-responsive factor, *GmARF11* forms a regulatory module that links nitrogen availability to the auxin signaling network, thereby enabling nitrogen-dependent inhibition of *GmNPF6.8.* This model is consistent with the established role of nitrate transporters, such as *AtNPF6.3/AtNRT1.1* in coordinating nitrate auxin crosstalk [54]. Therefore, we propose that the function of the GmARF11-GmNPF6.8 module converts nitrogen availability into auxin-mediated transcriptional responses, thereby regulating root development. This hypothesis is supported by transcriptional changes observed in *GmNPF6.8-*overexpressing lines, which show downregulation of growth-promoting genes (*GmEXP1*, *GmEXPB2*, *GmIDL4a*) and upregulation of the inhibitory transcription factor *GmERF57* (Fig. 7I). These results indicate that *GmNPF6.8* regulates root architecture through transcriptional reprogramming of key developmental regulators. In summary, the evolutionary, expression, and functional analyses of the *GmNPF* gene family reveal that *GmNPF6.8* is a central regulator of soybean root development and a major target of artificial selection during domestication. Its role as a negative regulator of root growth, combined with its near fixation in cultivated varieties, underscores its potential as a strategic target for improving nitrogen use efficiency in soybean breeding programs.

While this study provides strong evidence that *GmNPF6.8* acts as a negative regulator of nitrogen-responsive root development, its direct biochemical role as a nitrate transporter has yet to be experimentally confirmed. Current functional inferences rely on a chain of indirect evidence, including high sequence homology with the well-characterized nitrate transporter *AtNPF6.3/AtNRT1.1*, confirmed plasma membrane localization, nitrogen-responsive expression patterns, and consistent phenotypic effects in *Arabidopsis* overexpression lines. Direct functional verification remains a key focus for future research. Planned studies include assessing its transport kinetics in heterologous systems such as oocytes or yeast, and generating soybean mutants via CRISPR-Cas9 to test nitrate uptake in vivo using short-term ^15^N-labeled experiments. Elucidating the precise transport mechanism and physiological function of *GmNPF6.8* will provide definitive evidence for its role and establish a solid theoretical basis for its potential as a target to improve nitrogen use efficiency in soybean.

## Conclusion

This study provides a comprehensive genomic overview of the 126 *NPF* family members in soybean and identified *GmNPF6.8* as a major target of artificial selection during domestication. This conclusion is supported by the dramatic increase in the frequency of its major haplotype *GmNPF6.8*^*Hap1*^, from wild soybeans to improved cultivars. Functional analyses show that *GmNPF6.8* acts as a negative regulator of root development, reducing both lateral root density and primary root length. Moreover, *GmARF11* specifically binds to the promoter of the *GmNPF6.8* promoter, repressing its transcription. Together, these findings highlight *GmNPF6.8* as a promising candidate for genetic improvement of root architecture to enhance nitrogen use efficiency in soybean.

## Supplementary Information

Supplementary Material 1. Supplementary Figure S1: Analysis of cis-regulatory elements in the promoter of soybean *NPF* genes. Schematic diagram of cis-regulatory elements in the *GmNPF* genes promoter. Supplementary Figure S2: The frequency of representative cis-regulatory elements in the *GmNPF* genes promoter. Supplementary Figure S3: Expression profiles of the *GmNPF* genes in various tissues during soybean development. The heatmap shows transcript abundance (Log2(FPKM)) of *GmNPF* genes across various tissues at different developmental stages (DAF: days after flowering). Supplementary Figure S4: Analyzing the expression patterns of *GmNPF* genes under different nitrogen treatment conditions based on RNA-seq data. The scale bar represents the Log2 standardized expression. Supplementary Figure S5: Phylogenetic tree of NPF proteins from soybean and *Arabidopsis thaliana*. The unrooted neighbor-joining (NJ) tree was constructed using MEGA 7.0 with 1000 bootstrap replicates. Distinct subfamilies are highlighted with different colors, and the bootstrap confidence values are displayed on the branches. Supplementary Figure S6: Phylogenetic tree and subgroup classification of NPF proteins in *Glycine max*. Supplementary Figure S7: Analysis of amino acid sequence alignment between *GmNPF6.8* and *AtNPF6.3/AtNRT1.1*, with the red box indicating the PTR2 core region. Supplementary Figure S8: Phylogenetic relationships, gene structures, and conserved motifs among the 126 *GmNPF*s. A Phylogenetic tree constructed based on the full-length *GmNPF* protein sequences using MEGA 7.0. B Distribution of 10 conserved motifs, indicated by colored boxes. Sequence logos for each motif are shown in the upper right corner. The horizontal line at the bottom represents a protein length scale bar. C Gene model of *GmNPF* family members. Exons and untranslated regions (UTRs) are depicted by yellow and green boxes, respectively. Supplementary Figure S9: Sequence logo of the *GmNPFs* conserved-domain. Supplementary Figure S10: Natural variation and evolutionary analysis of the *GmNPF6*.*9* gene in soybean. A Identification of three major haplotypes (Hap1, Hap2, Hap3) in the *GmNPF6*.*9* coding region, defined by fourteen polymorphic sites. UTRs and exons are indicated by green and blue boxes, respectively. B Evolutionary analysis of three haplotypes in the coding region of *GmNPF6*.*9* gene. Supplementary Figure S11: Natural variation and evolutionary analysis of the *GmNPF2*.*11* gene in soybean. A Identification of three major haplotypes (Hap1, Hap2, Hap3) in the *GmNPF2.11* coding region, defined by five polymorphic sites. UTRs and exons are indicated by green and blue boxes, respectively. B Evolutionary analysis of three haplotypes in the coding region of* GmNPF2*.*11* gene. Supplementary Figure S12: Natural variation and evolutionary analysis of the *GmNPF5*.*13* gene in soybean. A Identification of six major haplotypes (Hap1, Hap2, Hap3, Hap4, Hap5, Hap6) in the *GmNPF5*.*13* coding region, defined by seven polymorphic sites. UTRs and exons are indicated by green and blue boxes, respectively. B Evolutionary analysis of six haplotypes in the coding region of *GmNPF5.13* gene. Supplementary Figure S13: Natural variation and evolutionary analysis of the *GmNPF7*.*13 *gene in soybean. A Identification of six major haplotypes (Hap1, Hap2) in the *GmNPF7*.*13* coding region, defined by two polymorphic sites. UTRs and exons are indicated by green and blue boxes, respectively. B Evolutionary analysis of two haplotypes in the coding region of *GmNPF7.13* gene. Supplementary Figure S14: Relative expression levels of *GmNPF6*.*8* in roots, nodules, stems, leaves, and flowers of soybean cultivar W82, as determined by qRT-PCR. Supplementary Figure S15: Phylogenetic tree of *GmARF11*, *NtARF11*, and *Arabidopsis* ARF protein. The unrooted neighbor-joining (NJ) tree was constructed using MEGA 7.0 with 1000 bootstrap replicates.

Supplementary Material 2. Supplementary Table S1: Information of 126 *NPF* genes identified in the Wm82.a4.v1 reference genome (V4).

Supplementary Material 3. Supplementary Table S2: Gene density of each Chromosome of the soybean genome.

Supplementary Material 4. Supplementary Table S3: Tandemly and segmentally duplicated *GmNPF* gene pairs.

Supplementary Material 5. Supplementary Table S4: One-to-one orthologous relationships between the *GmNPF* gene members in *Glycine* max.

Supplementary Material 6.Supplementary Table S5: One-to-one orthologous relationships between the *NPF* gene members in *Glycine max* and *Oryza sativa, Arabidopsis thaliana, Solanum lycopersicum, Vigna unguiculata*.

Supplementary Material 7. Supplementary Table S6: Analysis of cis-regulatory elements in the 2000 bp promoter region of soybean *NPF* genes using the New PLACE database.

Supplementary Material 8. Supplementary Table S7: Expression profiles of *GmNPF* genes in multiple tissues throughout various developmental stages. Supplementary Table S8: Expression profiles of *GmNPF* genes family under N deficiency treatments. Supplementary Table S9: List of PCR primers used in this study.

Supplementary Material 9. Supplementary Table S8: Expression profiles of *GmNPF* genes family under N deficiency treatments. Supplementary Table S9: List of PCR primers used in this study.

Supplementary Material 10. Supplementary Table S9: List of PCR primers used in this study.

Supplementary Material 11. Supplementary Table S10: Summary of key *GmNPF* genes with distinct tissue-specific expression patterns. Supplementary Table S11: Details of the 10 conserved motifs of soybean NPF proteins. Supplementary Table S12: Analysis of *GmNPF* genes with significant differential expression in nitrogen response. Supplementary Table S13: Sequence variations among *GmNPF6.8* haplotypes.

## Data Availability

The datasets supporting the conclusions of this article are included within the article and its additional files. And the transcriptome data analyzed during the current study are available in the Plant Public RNA-seq Database (PPRD, http://ipf.sustech.edu.cn/pub/plantrna/. Accession numbers: PRJNA662487).
