# Supplementary material for: Comprehensive genome-wide identification of the NPF gene family and functional characterization of GmNPF6.8 regulating root development in soybean
Source: BMC Plant Biol. 2026 Mar 17;26:748. doi: 10.1186/s12870-026-08559-x (PMC13107649; doi:10.1186/s12870-026-08559-x)
Supplement: Supplementary file 1 — Supplementary Material 1. Supplementary Figure S1: Analysis of cis-regulatory elements in the promoter of soybean NPF genes. Schematic diagram of cis-regulatory elements in the GmNPF genes promoter. Supplementary Figure S2: The frequency of representative cis-regulatory elements in the GmNPF genes promoter. Supplementary Figure S3: Expression profiles of the GmNPF genes in various tissues during soybean development. The heatmap shows transcript abundance (Log2(FPKM)) of GmNPF genes across various tissues at different developmental stages (DAF: days after flowering). Supplementary Figure S4: Analyzing the expression patterns of GmNPF genes under different nitrogen treatment conditions based on RNA-seq data. The scale bar represents the Log2 standardized expression. Supplementary Figure S5: Phylogenetic tree of NPF proteins from soybean and Arabidopsis thaliana. The unrooted neighbor-joining (NJ) tree was constructed using MEGA 7.0 with 1000 bootstrap replicates. Distinct subfamilies are highlighted with different colors, and the bootstrap confidence values are displayed on the branches. Supplementary Figure S6: Phylogenetic tree and subgroup classification of NPF proteins in Glycine max. Supplementary Figure S7: Analysis of amino acid sequence alignment between GmNPF6.8 and AtNPF6.3/AtNRT1.1, with the red box indicating the PTR2 core region. Supplementary Figure S8: Phylogenetic relationships, gene structures, and conserved motifs among the 126 GmNPFs. A Phylogenetic tree constructed based on the full-length GmNPF protein sequences using MEGA 7.0. B Distribution of 10 conserved motifs, indicated by colored boxes. Sequence logos for each motif are shown in the upper right corner. The horizontal line at the bottom represents a protein length scale bar. C Gene model of GmNPF family members. Exons and untranslated regions (UTRs) are depicted by yellow and green boxes, respectively. Supplementary Figure S9: Sequence logo of the GmNPFs conserved-domain. Supplementary Figur [file 12870_2026_8559_MOESM1_ESM.docx]

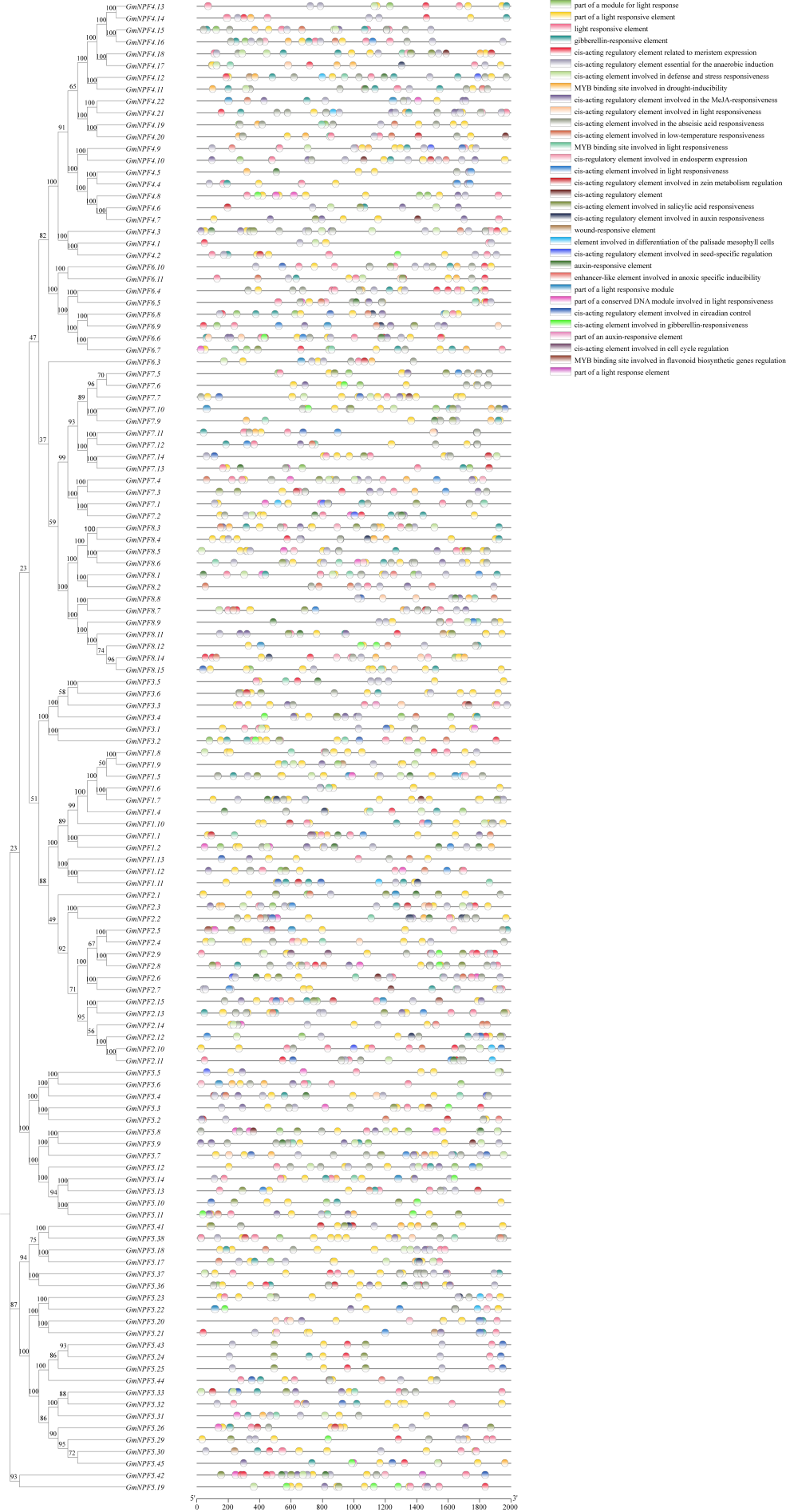


**Supplementary Figure S1.** Analysis of cis-regulatory elements in the promoter of soybean *NPF* genes. Schematic diagram of cis elements in the *GmNPF* genes promoter.


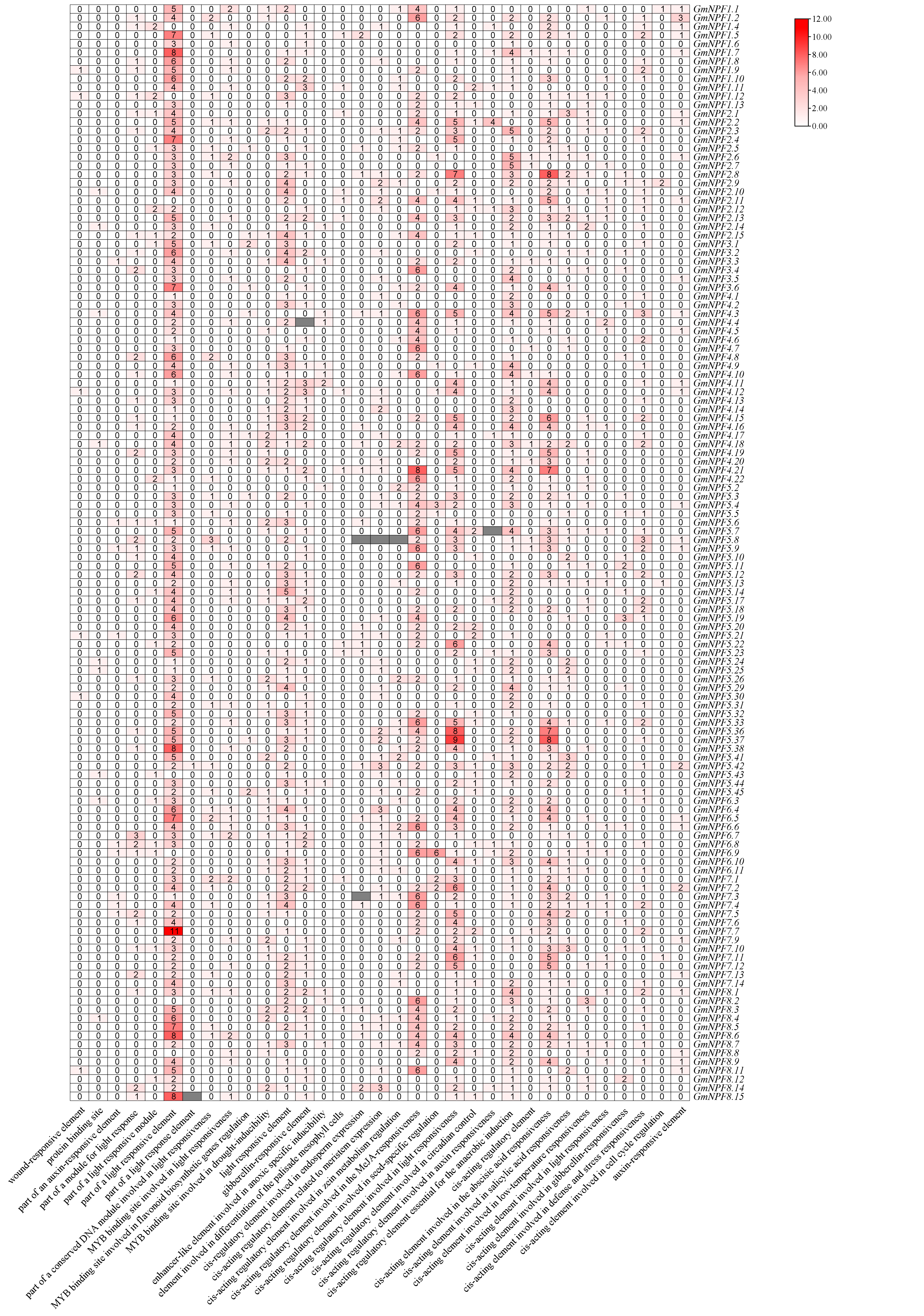


**Supplementary Figure S2.** The frequency of representative cis-acting elements in the *GmNPF* genes promoter.


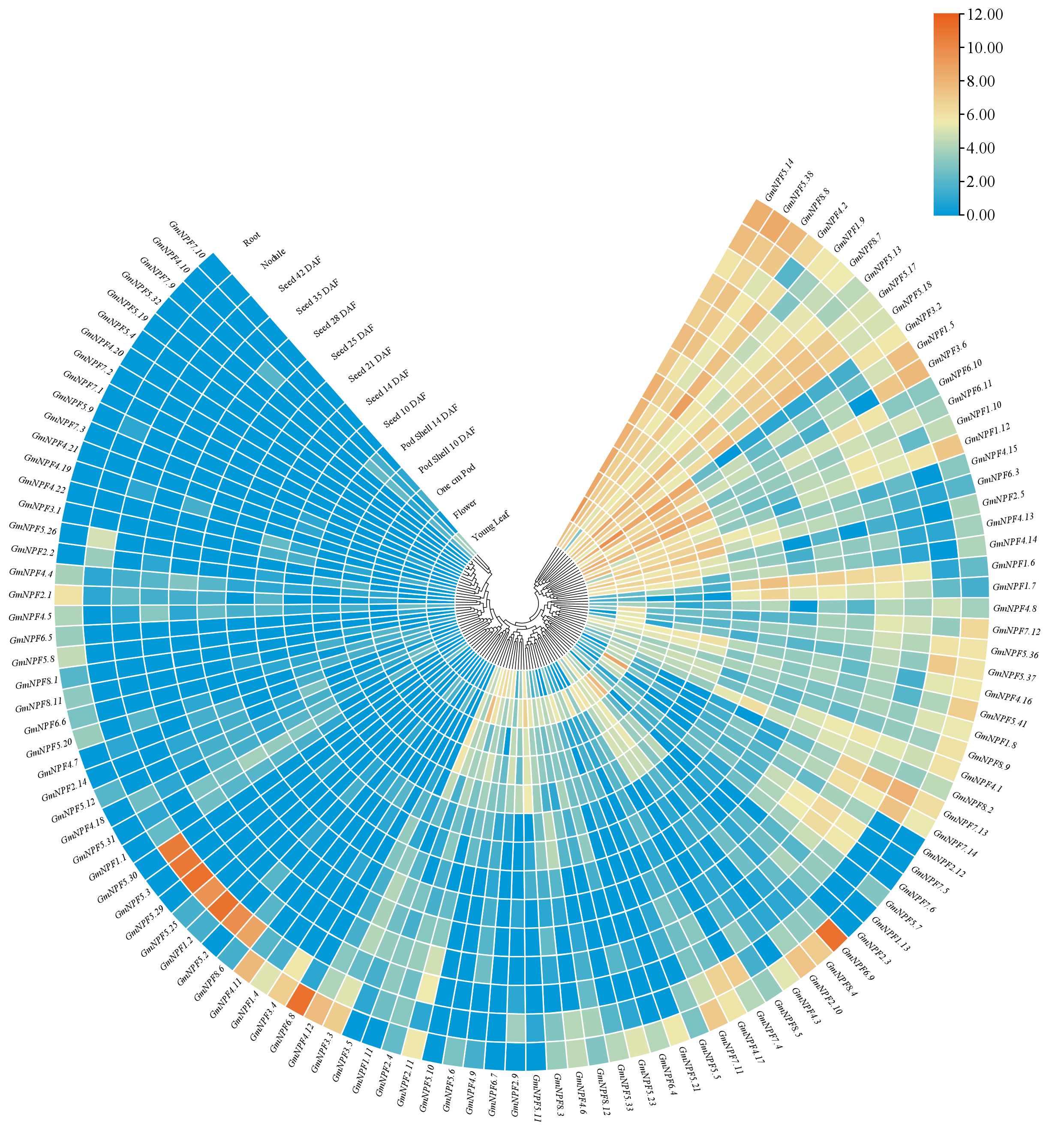


**Supplementary Figure S3.** Expression profiles of the *GmNPF* genes in various tissues during soybean development. The heatmap shows transcript abundance (Log_2_(FPKM)) of *GmNPF* genes across various tissues at different developmental stages (DAF: days after flowering).


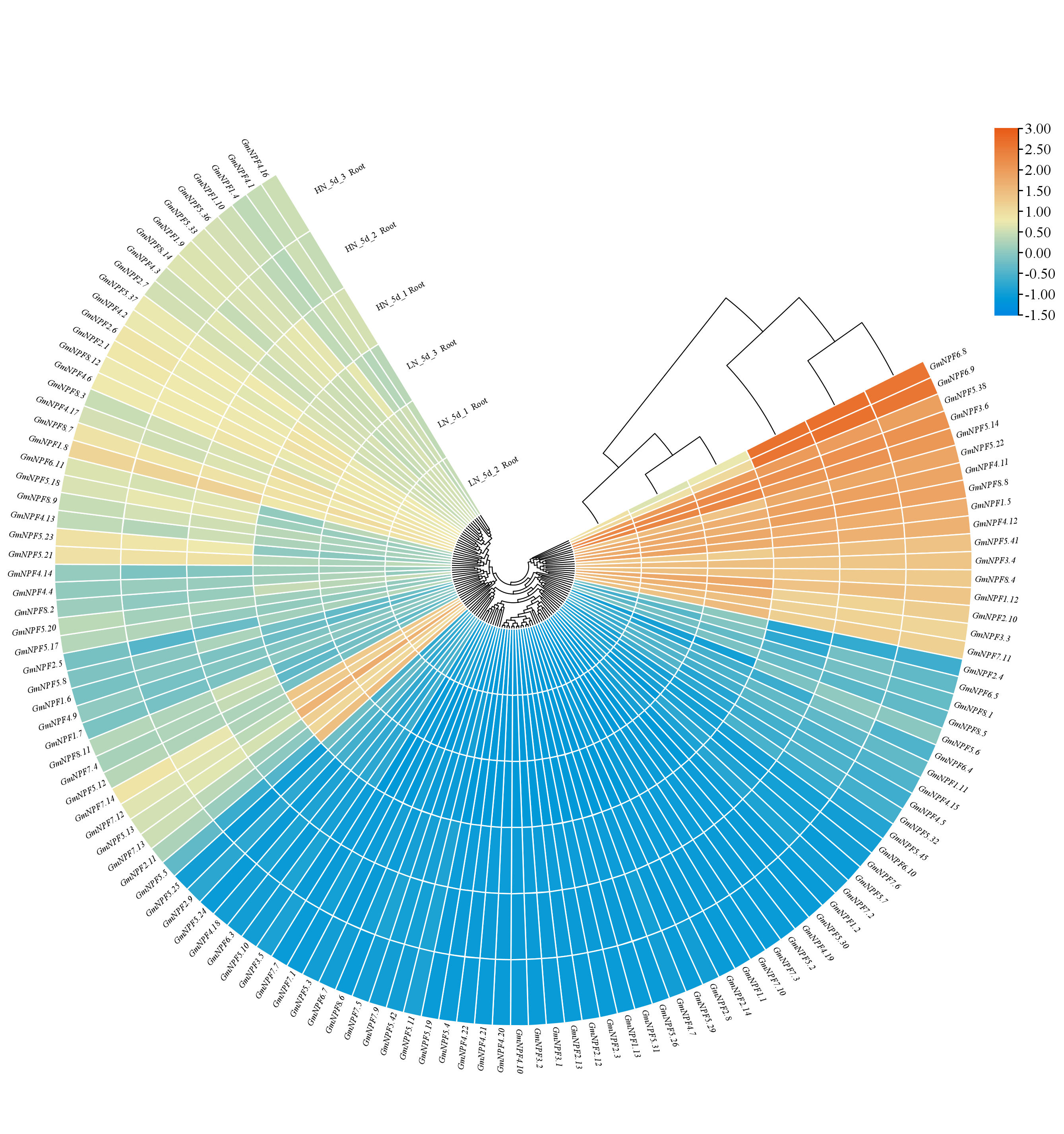


**Supplementary Figure S4.** Analyzing the expression patterns of *GmNPF* genes under different nitrogen treatment conditions based on RNA-seq data. The scale bar represents the Log_2_ standardized expression.


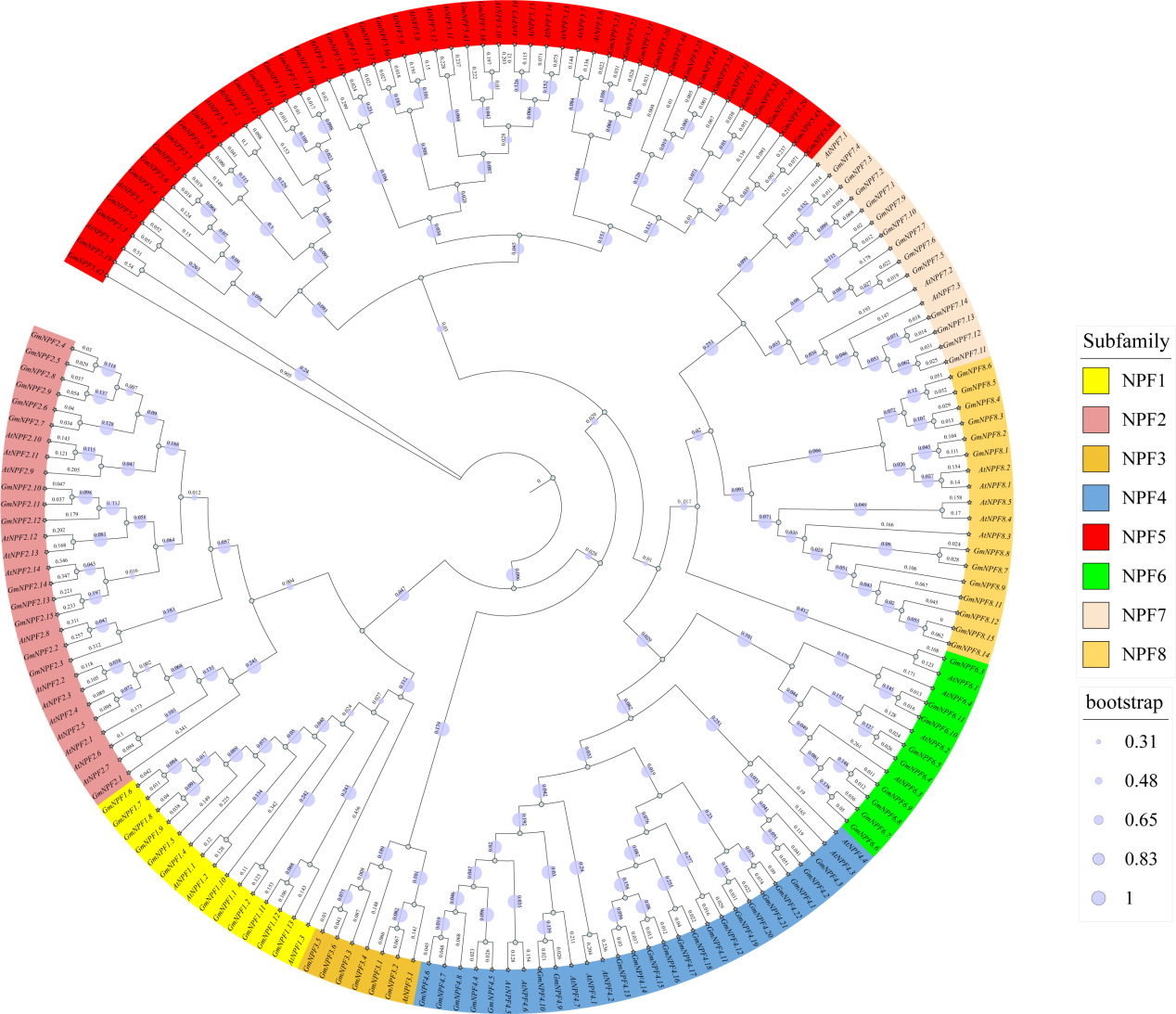


**Supplementary Figure S5.** Phylogenetic tree of NPF proteins from soybean and *Arabidopsis thaliana*. The unrooted neighbor-joining (NJ) tree was constructed using MEGA7.0 with 1000 bootstrap replicates. Distinct subfamilies are highlighted with different colors, and the bootstrap confidence values are displayed on the branches.


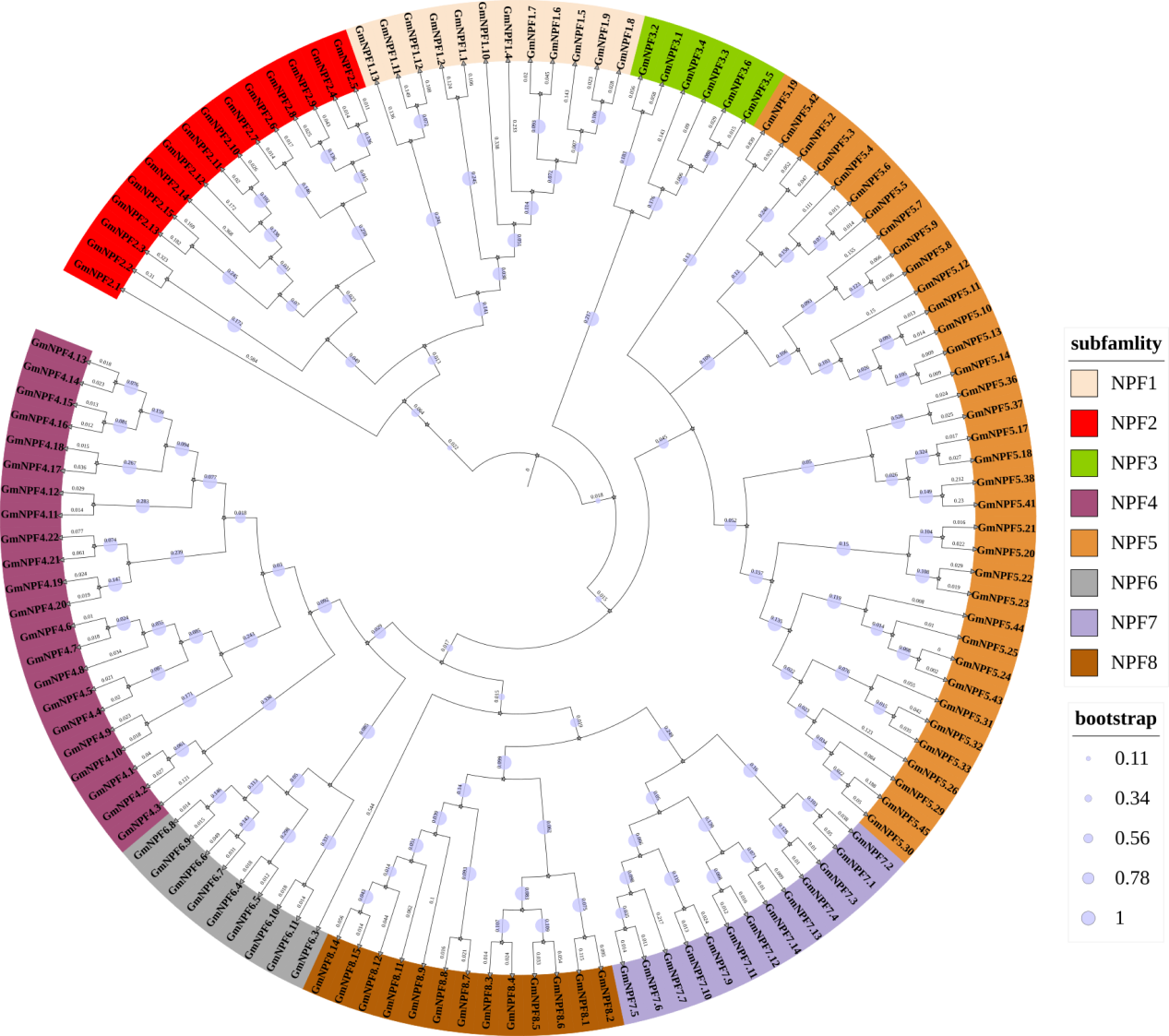


**Supplementary Figure S6.** Phylogenetic tree and subgroup classification of NPF proteins in *Glycine max*.

**
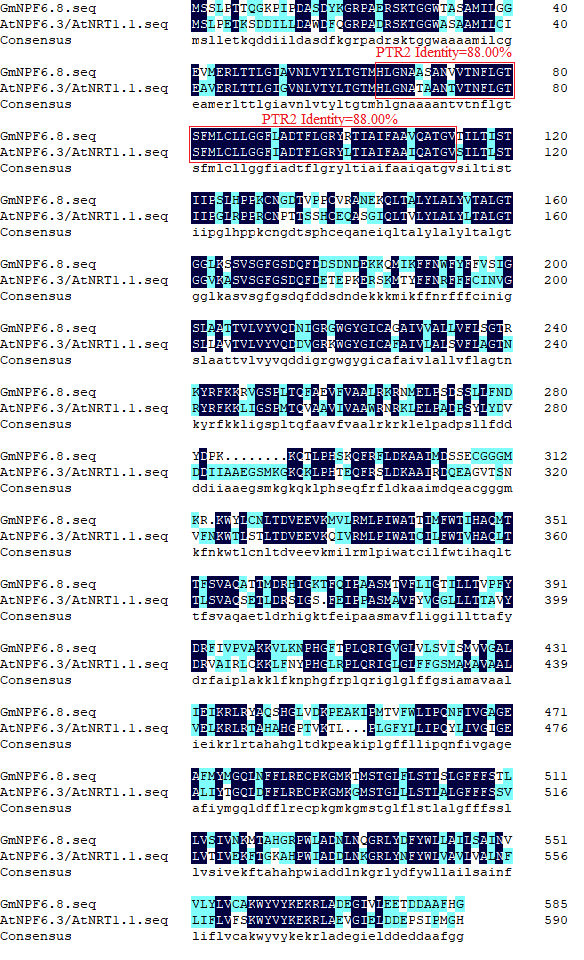
**

**Supplementary Figure S7.** Analysis of amino acid sequence alignment between *GmNPF6.8* and *AtNPF6.3/AtNRT1.1*, with the red box indicating the PTR2 core region.

**
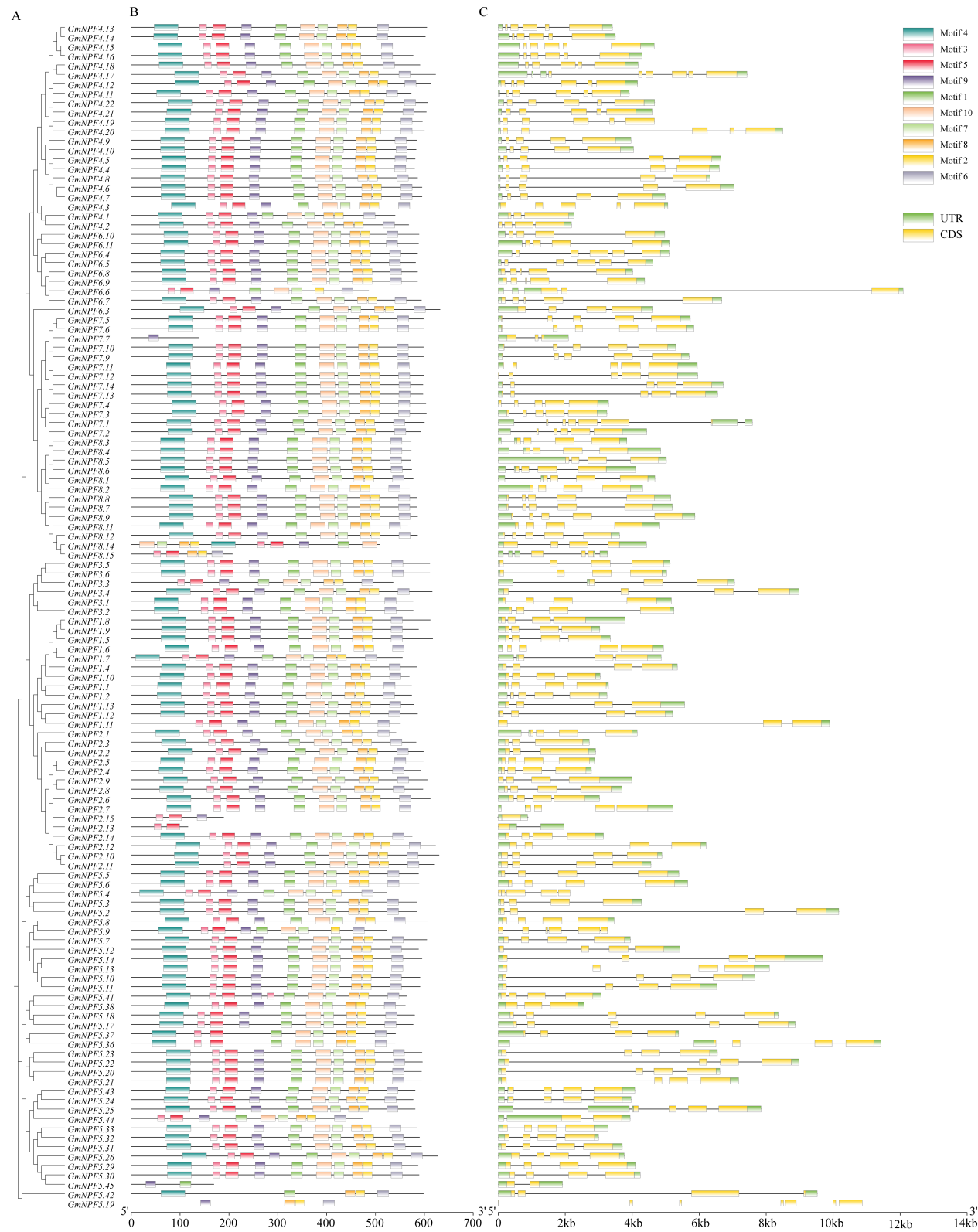
**

**Supplementary Figure S8.** Phylogenetic relationships, gene structures, and conserved motifs among the 126 *GmNPFs*. **A** Phylogenetic tree constructed based on the full-length GmNPF protein sequences using MEGA 7.0. **B** Distribution of 10 conserved motifs, indicated by colored boxes. Sequence logos for each motif are shown in the upper right corner. The horizontal line at the bottom represents a protein length scale bar. **C** Gene model of *GmNPF* family members. Exons and untranslated regions (UTRs) are depicted by yellow and green boxes, respectively.

**
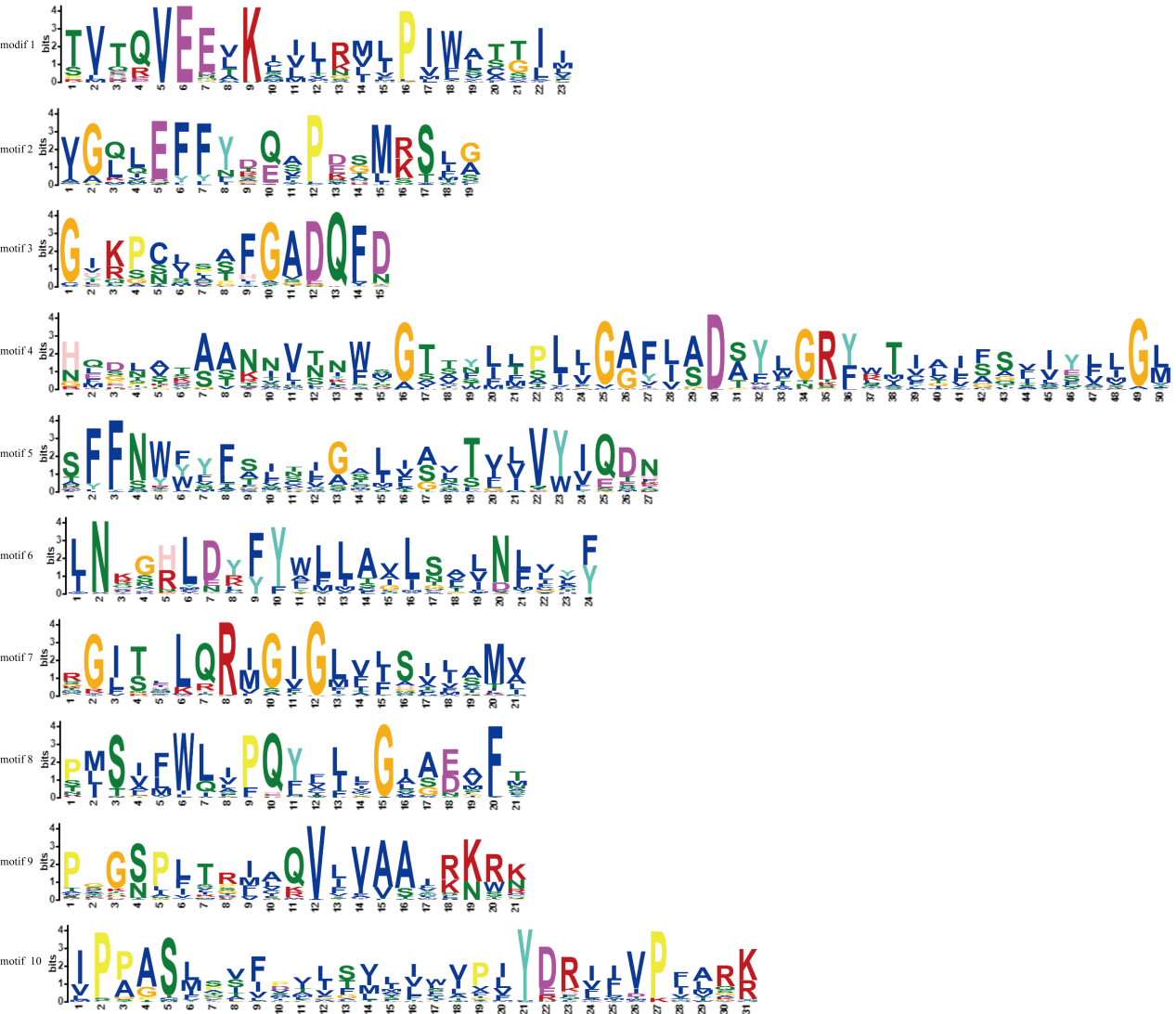
**

**Supplementary Figure S9.** Sequence logo of the *GmNPFs* conserved-domain.


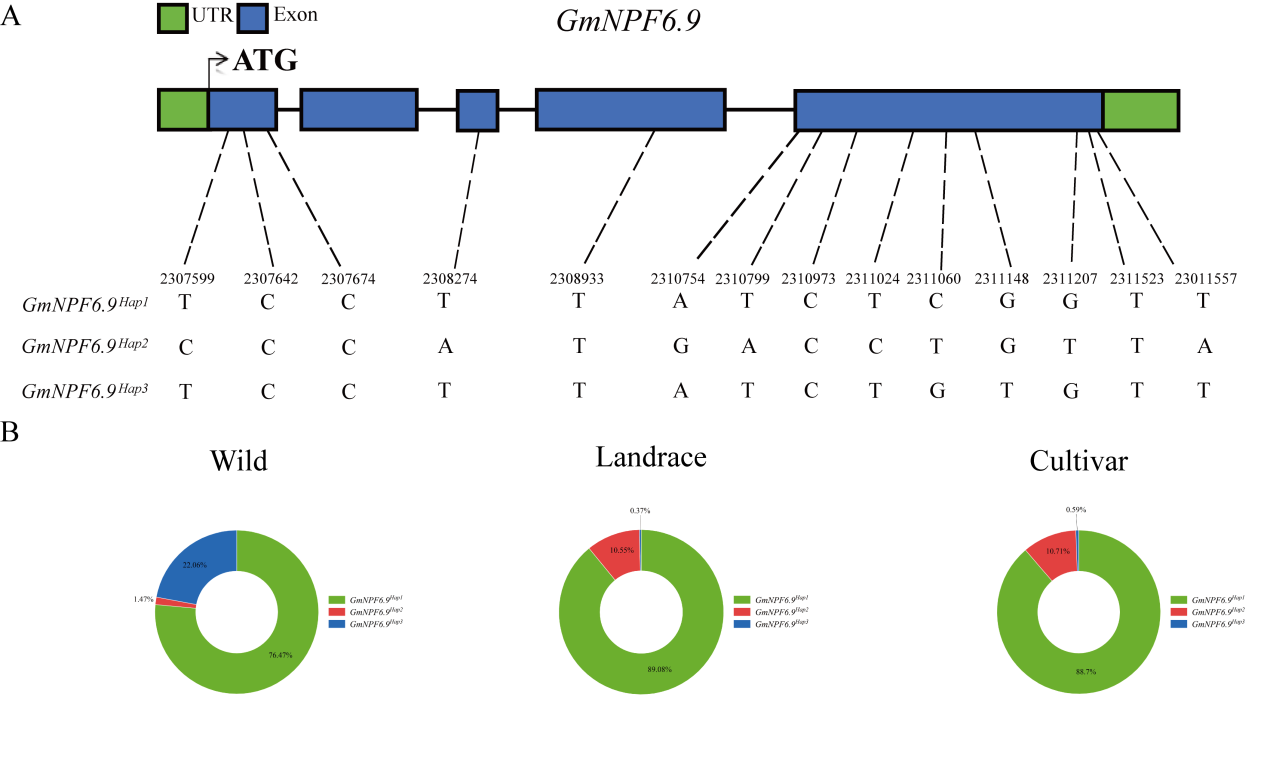


**Supplementary Figure S10.** Natural variation and evolutionary analysis of the *GmNPF6.9* gene in soybean. **A** Identification of three major haplotypes (Hap1, Hap2, Hap3) in the *GmNPF6.9* coding region, defined by fourteen polymorphic sites. UTRs and exons are indicated by green and blue boxes, respectively. **B** Evolutionary analysis of three haplotypes in the coding region of *GmNPF6.9* gene.


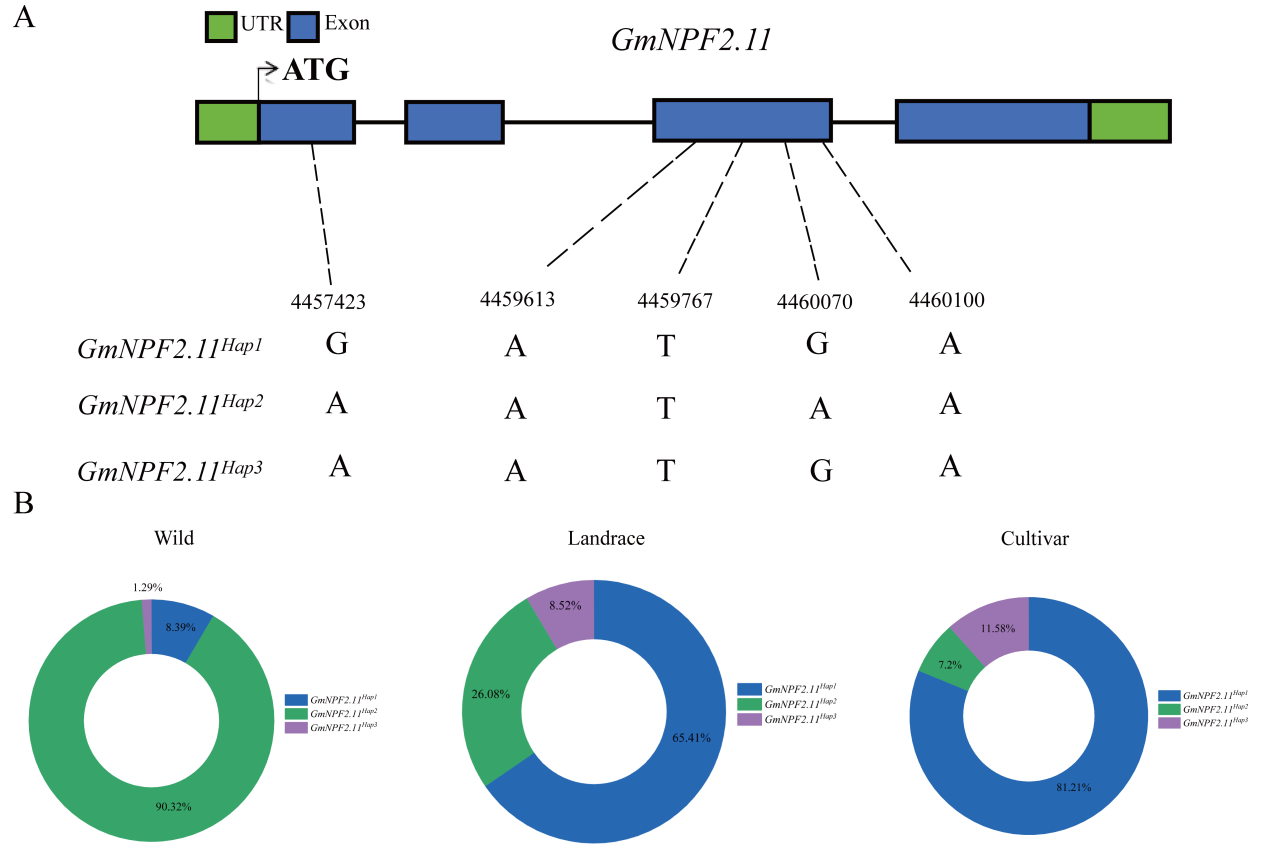


**Supplementary Figure S11.** Natural variation and evolutionary analysis of the *GmNPF2.11* gene in soybean. **A** Identification of three major haplotypes (Hap1, Hap2, Hap3) in the *GmNPF2.11* coding region, defined by five polymorphic sites. UTRs and exons are indicated by green and blue boxes, respectively. **B** Evolutionary analysis of three haplotypes in the coding region of *GmNPF2.11* gene.


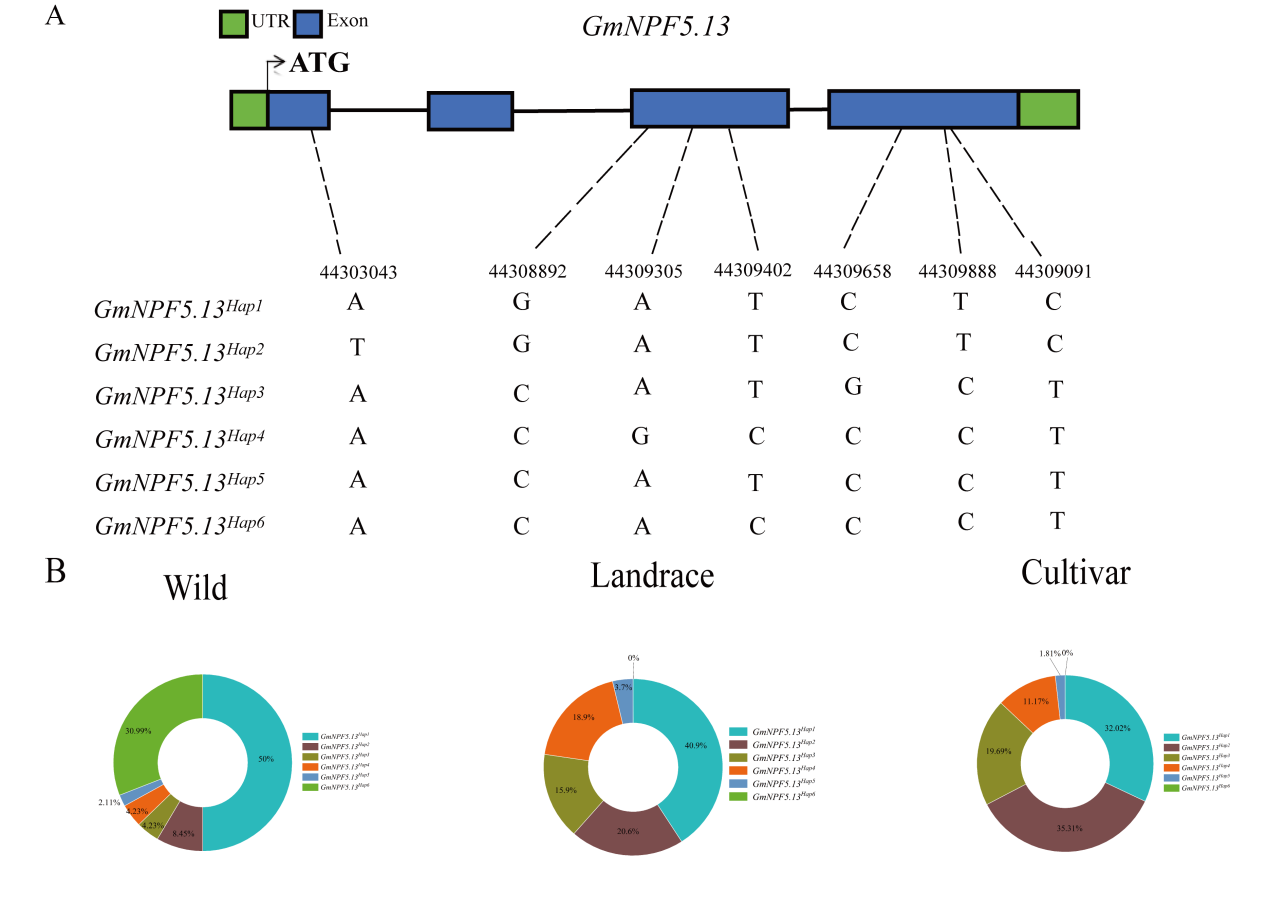


**Supplementary Figure S12.** Natural variation and evolutionary analysis of the *GmNPF5.13* gene in soybean. **A** Identification of six major haplotypes (Hap1, Hap2, Hap3, Hap4, Hap5, Hap6) in the *GmNPF5.13* coding region, defined by seven polymorphic sites. UTRs and exons are indicated by green and blue boxes, respectively. **B** Evolutionary analysis of six haplotypes in the coding region of *GmNPF5.13* gene.

**
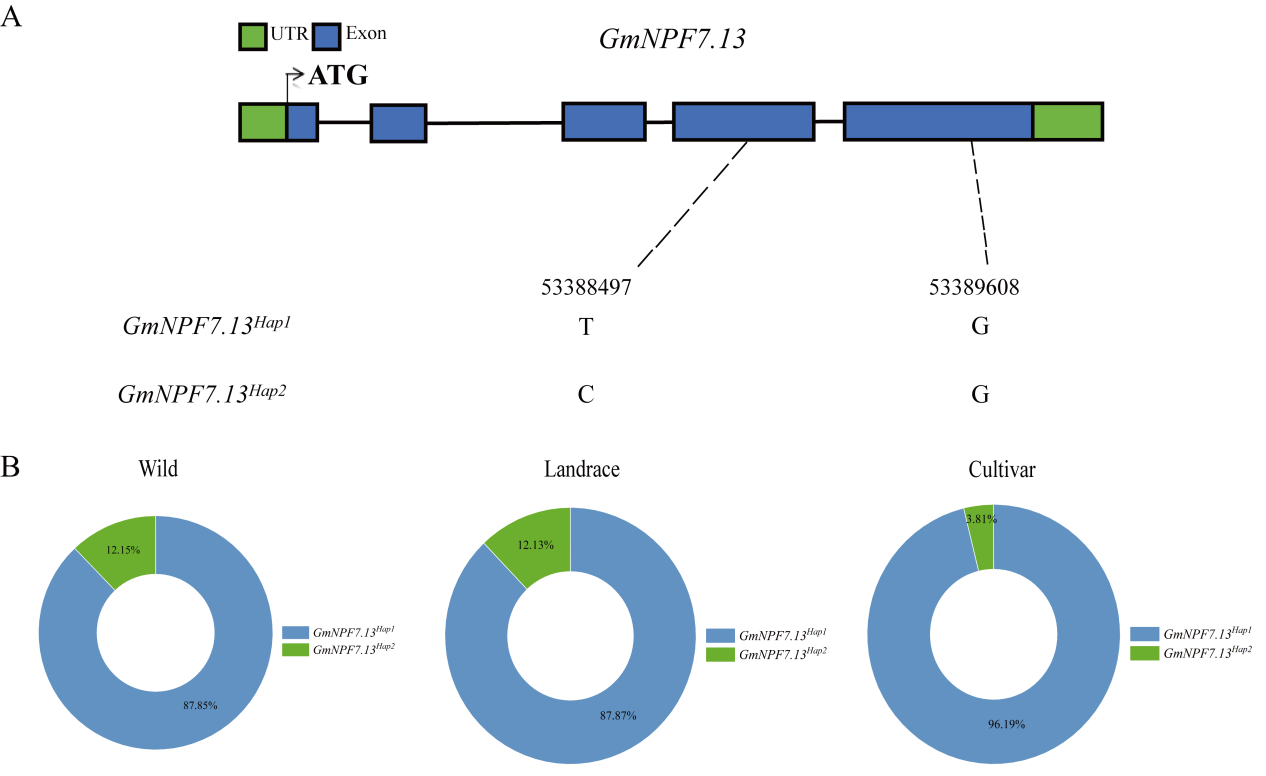
**

**Supplementary Figure S13.** Natural variation and evolutionary analysis of the *GmNPF7.13* gene in soybean. **A** Identification of six major haplotypes (Hap1, Hap2) in the *GmNPF7.13* coding region, defined by two polymorphic sites. UTRs and exons are indicated by green and blue boxes, respectively. **B** Evolutionary analysis of two haplotypes in the coding region of *GmNPF7.13* gene.

**
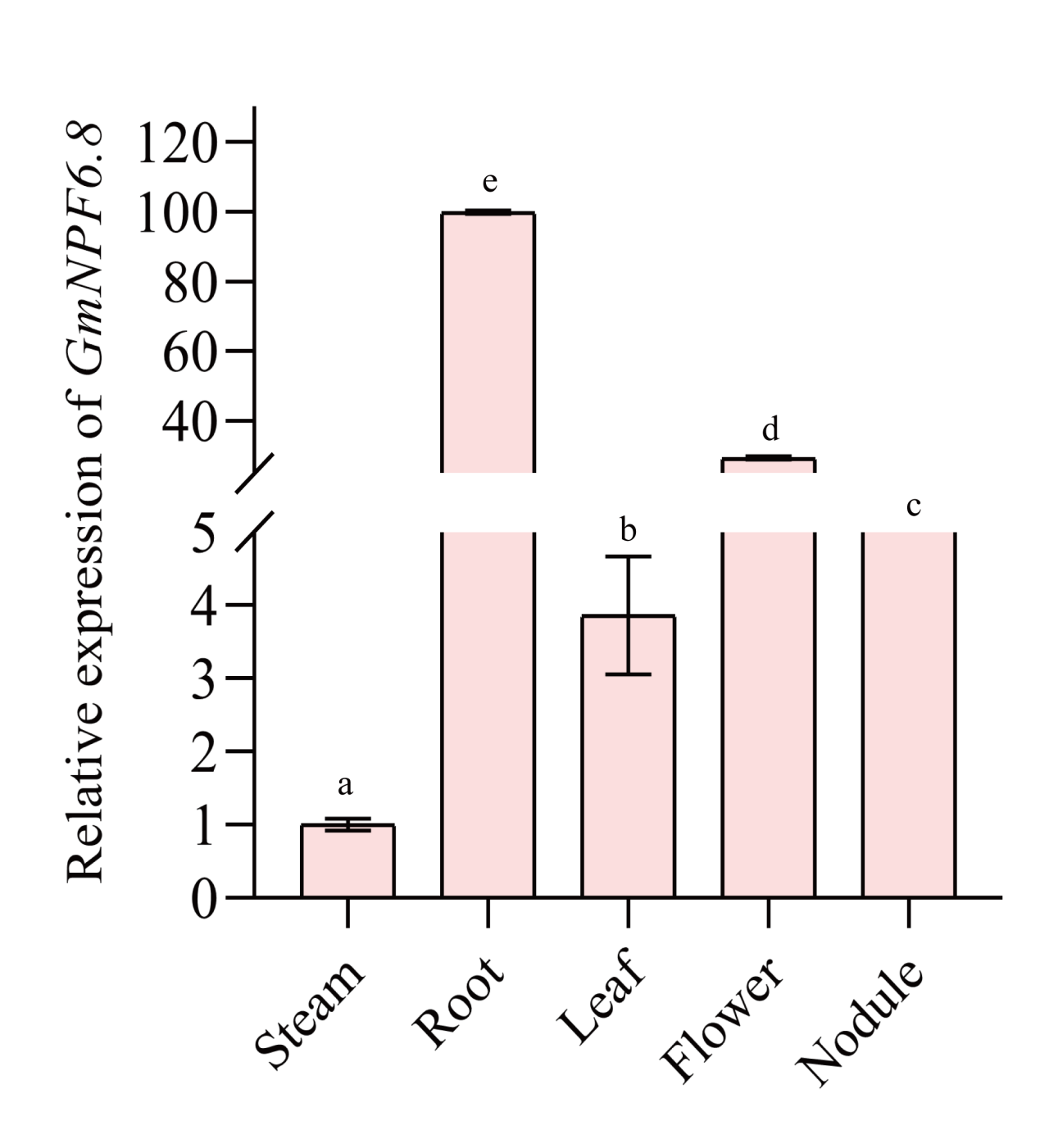
**

**Supplementary Figure S14.** Relative expression levels of *GmNPF6.8* in roots, nodules, stems, leaves, and flowers of soybean cultivar W82, as determined by qRT-PCR.

**
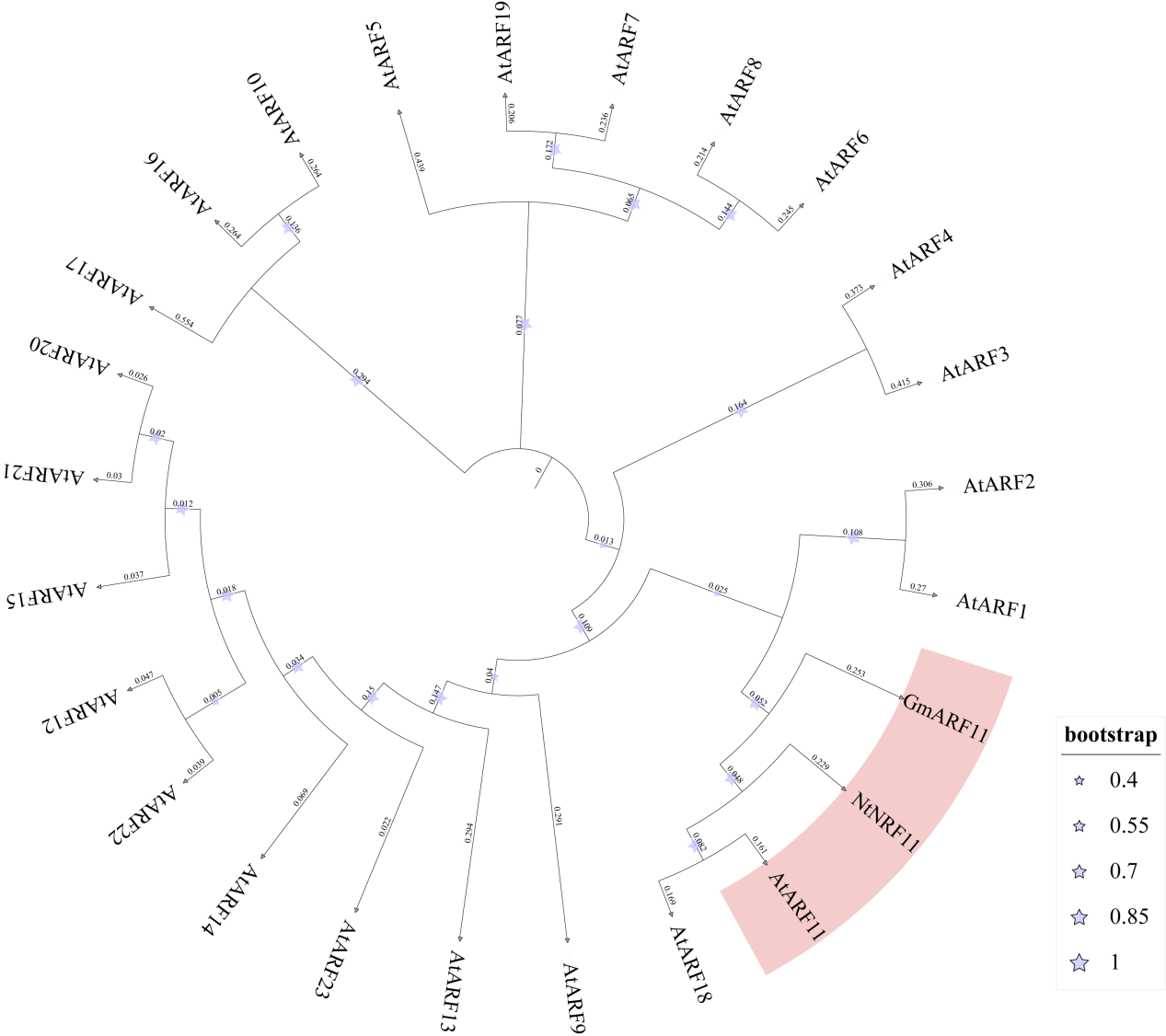
**

**Supplementary Figure S15.** Phylogenetic tree of *GmARF11*, *NtARF11*, and *Arabidopsis* ARF protein. The unrooted neighbor-joining (NJ) tree was constructed using MEGA7.0 with 1000 bootstrap replicates.
