# Supplementary material for: Comprehensive genome-wide identification of the NPF gene family and functional characterization of GmNPF6.8 regulating root development in soybean
Source: BMC Plant Biol. 2026 Mar 17;26:748. doi: 10.1186/s12870-026-08559-x (PMC13107649; doi:10.1186/s12870-026-08559-x)
Supplement: Supplementary file 11 — Supplementary Material 11. Supplementary Table S10: Summary of key GmNPF genes with distinct tissue-specific expression patterns. Supplementary Table S11: Details of the 10 conserved motifs of soybean NPF proteins. Supplementary Table S12: Analysis of GmNPF genes with significant differential expression in nitrogen response. Supplementary Table S13: Sequence variations among GmNPF6.8 haplotypes. [file 12870_2026_8559_MOESM11_ESM.docx]

**Supplementary Table 10** Summary of key *GmNPF* genes with distinct tissue-specific expression patterns.

| Gene Name | Young Leaf | Flower | One cm Pod | Seed 10 DAF | Nodule | Root | Expression Category | Putative Function |
| --- | --- | --- | --- | --- | --- | --- | --- | --- |
| *GmNPF1.2* | 0 | 0 | 0 | 0 | 2154 | 2 | Nodule-Specific Expression | Potential involvement in nodule development and function. |
| *GmNPF5.2* | 0 | 0 | 0 | 1 | 907 | 0 |  |  |
| *GmNPF5.3* | 4 | 0 | 0 | 0 | 1755 | 0 |  |  |
| *GmNPF8.6* | 0 | 4 | 0 | 0 | 440 | 2 |  |  |
| *GmNPF5.25* | 0 | 1 | 0 | 0 | 758 | 2 |  |  |
| *GmNPF5.29* | 0 | 0 | 0 | 0 | 2331 | 0 |  |  |
| *GmNPF5.30* | 1 | 0 | 0 | 0 | 1542 | 0 |  |  |
| *GmNPF5.38* | 109 | 176 | 128 | 81 | 140 | 367 | Constitutive High Expression | Housekeeping roles in fundamental soybean development processes. |
| *GmNPF5.13* | 171 | 386 | 273 | 135 | 14 | 20 |  |  |
| *GmNPF5.14* | 197 | 647 | 402 | 262 | 207 | 313 |  |  |
| *GmNPF6.8* | 1 | 4 | 0 | 0 | 1 | 3342 | Root-Specific Expression | Potential key regulators in root-related processes. |
| *GmNPF6.9* | 17 | 232 | 3 | 0 | 7 | 2282 |  |  |

Note: Select one cm pod and Seed 10 DAF for organizational expression display in the table.

**Supplementary Table 11**  Details of the 10 conserved motifs of soybean NPF proteins.

| Motif | Width(aa) | Motif sequence |
| --- | --- | --- |
| Motif1 | 23 | TVTQVEEVKCILRLLPIWLCTIM |
| Motif2 | 19 | VGQLEFFYDQSPDAMRSLC |
| Motif3 | 15 | GHKPCVQAFGADQFD |
| Motif4 | 50 | NQHSATASKNVSNWSGTCYITPLIGAYLADSYLGRYWTIAVFSIIYAIGM |
| Motif5 | 27 | SFFNWWMFSIFIGTLFANSVLVYIQDN |
| Motif6 | 24 | LNRGHLDYFYWLLTVLSFLNFLVY |
| Motif7 | 21 | MGITHLQRIGIGLVLSIVAMA |
| Motif8 | 21 | PISVFWLVPQYCLHGLADVFM |
| Motif9 | 21 | PGGSPLTRVAQVLVAAFRKRN |
| Motif10 | 31 | IPPASLSSFDVISVIVWVPVYDRIIVPIARK |

**Supplementary Table 12** Analysis of *GmNPF* genes with significant differential expression in nitrogen response.

| Gene Name | HN (Expression Average) | LN (Expression Average) | Log_2_(LN/HN) | *P*-value | Gene expression (Up/Down) |
| --- | --- | --- | --- | --- | --- |
| *GmNPF5.18* | 7.30 | 3.50 | -1.06 | *P*<0.01 | Down |
| *GmNPF5.21* | 10.23 | 4.73 | -1.11 | *P*<0.01 | Down |
| *GmNPF6.8* | 92.37 | 9.23 | -3.32 | *P*<0.01 | Down |
| *GmNPF6.9* | 88.60 | 12.67 | -2.81 | *P*<0.01 | Down |
| *GmNPF5.6* | 2.43 | 0.20 | -3.60 | *P*<0.01 | Down |
| *GmNPF5.23* | 9.50 | 3.10 | -1.62 | *P*<0.01 | Down |
| *GmNPF7.4* | 4.30 | 1.70 | -1.34 | *P*<0.01 | Down |
| *GmNPF8.11* | 4.73 | 1.97 | -1.27 | *P*<0.01 | Down |
| *GmNPF2.4* | 0.50 | 2.53 | 2.34 | *P*<0.01 | Up |
| *GmNPF2.10* | 14.40 | 28.60 | 0.99 | *P*<0.01 | Up |
| *GmNPF2.11* | 3.20 | 13.43 | 2.07 | *P*<0.01 | Up |
| *GmNPF7.12* | 6.33 | 16.37 | 1.37 | *P*<0.01 | Up |
| *GmNPF7.13* | 4.70 | 15.83 | 1.75 | *P*<0.01 | Up |
| *GmNPF2.5* | 1.53 | 4.20 | 1.45 | *P*<0.01 | Up |
| *GmNPF5.5* | 1.00 | 22.27 | 4.48 | *P*<0.01 | Up |
| *GmNPF3.3* | 13.60 | 23.26 | 0.77 | *P*<0.01 | Up |
| *GmNPF5.13* | 6.60 | 29.57 | 2.16 | *P*<0.01 | Up |

Note: HN:High Nitrogen; LN:Low Nitrogen.

Supplementary Table 13 Sequence variations among *GmNPF6.8* haplotypes.

| Comparison of different haplotypes | SNP Location | SNP Mutation | Amino Acid Mutation | Non/Synonymous Mutation |
| --- | --- | --- | --- | --- |
| *GmNPF6.8^Hap1^*  vs  *GmNPF6.8^Hap2^* | 54185547 | T → T | Phe → Phe | No Mutation |
|  | 54186102 | G → G | Ala → Ala | No Mutation |
|  | 54186117 | T → A | Ala → Ala | Synonymous Mutation |
|  | 54186165 | C → C | Asp → Asp | No Mutation |
|  | 54186435 | G → T | Leu → Leu | Synonymous Mutation |
|  | 54188051 | G → G | Arg → Arg | No Mutation |
|  | 54188069 | A → A | Asn → Asn | No Mutation |
|  | 54188295 | C → T | Ile → Ile | Synonymous Mutation |
|  | 54188847 | T → T | Asp → Asp | No Mutation |
| *GmNPF6.8^Hap1^*  vs  *GmNPF6.8^Hap3^* | 54185547 | T → T | Phe → Phe | No Mutation |
|  | 54186102 | G → G | Ala → Ala | No Mutation |
|  | 54186117 | T → T | Ala → Ala | No Mutation |
|  | 54186165 | C → C | Asp → Asp | No Mutation |
|  | 54186435 | G → G | Leu → Leu | No Mutation |
|  | 54188051 | G → A | Arg → Lys | Non Synonymous Mutation |
|  | 54188069 | A → C | Asn → Thr | Non Synonymous Mutation |
|  | 54188295 | C → T | Ile → Ile | No Mutation |
|  | 54188847 | T → T | Asp → Asp | No Mutation |
